# Stiffness control in dual color tomographic volumetric 3D printing

**DOI:** 10.1038/s41467-022-28013-4

**Published:** 2022-01-18

**Authors:** Bin Wang, Einstom Engay, Peter R. Stubbe, Saeed Z. Moghaddam, Esben Thormann, Kristoffer Almdal, Aminul Islam, Yi Yang

**Affiliations:** 1 Department of Mechanical Engineering, Technical University of Denmark, 2800 Kongens Lyngby, Denmark; 2 National Center for Nano Fabrication and Characterization, Technical University of Denmark, 2800 Kongens Lyngby, Denmark; 3 National Food Institute, Technical University of Denmark, 2800 Kongens Lyngby, Denmark; 4 Department of Chemistry, Technical University of Denmark, 2800 Kongens Lyngby, Denmark; 5 Center for Energy Resources Engineering, Technical University of Denmark, 2800 Kongens Lyngby, Denmark

**Keywords:** Mechanical engineering, Mechanical properties, Polymers

## Abstract

Tomographic volumetric printing (TVP) physically reverses tomography to offer fast and auxiliary-free 3D printing. Here we show that wavelength-sensitive photoresins can be cured using visible ($\bar{\lambda }=455$ nm) and UV ($\bar{\lambda }=365$ nm) sources simultaneously in a TVP setup to generate internal mechanical property gradients with high precision. We develop solutions of mixed acrylate and epoxy monomers and utilize the orthogonal chemistry between free radical and cationic polymerization to realize fully 3D stiffness control. The radial resolution of stiffness control is 300 µm or better and an average modulus gradient of 5 MPa/µm is achieved. We further show that the reactive transport of radical inhibitors defines a workpiece’s shape and limits the achievable stiffness contrast to a range from 127 MPa to 201 MPa according to standard tensile tests after post-processing. Our result presents a strategy for controlling the stiffness of material spatially in light-based volumetric additive manufacturing.

## Abstract (web summary)

Tomographic volumetric printing (TVP) is considered as a fast and auxiliary-free 3D printing technique, but control of internal mechanical properties remains less investigated for TVP. Here, the authors show that wavelength-sensitive photoresins can be cured using visible and UV sources simultaneously in a TVP setup to generate internal mechanical property gradients with high precision.

## Introduction

Hierarchically branched structures are a hallmark of natural and biological systems^1^. Being able to recreate these multiscale structures in a synthetic system will, among other bio-inspired applications, pave the way to engineering fully vascularized artificial tissues with clinically relevant size^2,3^. Given the very high geometric complexity, it is tempting to use additive manufacturing (AM) techniques to create such structures^4–6^. However, building a multiscale structure requires an intrinsic 3D method^7^. If *n* is the number of voxels needed in one direction to represent a target geometry, the processing time of AM scales with *n*^3-*D*^, in which *D* is the intrinsic dimensionality of the AM method. For example, *D* = 0 for methods based on point-to-point deposition or writing^8,9^, 1 for line scanning^10^ and 2 for layer-by-layer stereolithography^11,12^. Although every single step in a lower-dimensional AM method can be fast, the total processing time stacks up quickly with sequential operations. *n* can be enormous for structures that demonstrate fractality on even only a few orders of magnitude, rendering the processing time impractically long for many applications when *D* < 3.

Recent advances in volumetric AM employed different strategies to increase printing speed^13–15^. In particular, computed axial lithography^16^ (or tomographic volumetric 3D printing, TVP) was shown to operate on a length scale relevant for tissue engineering^17^. TVP physically reverses computed tomography (CT) to create a 3D energy distribution in a photoresponsive curing volume. By projecting light patterns from different angles, TVP cures all points in an object in parallel, severing the dependence of printing time on voxel number. It is intrinsically 3D and has a reported resolution of up to ~80 μm^18^, with room for improvement. TVP can be several orders of magnitude faster than conventional AM methods and can be used with other microfabrication methods or existing objects (overprinting)^16^. When processing very soft hydrogel or suspended objects, TVP does not require auxiliary support because the geometric integrity of a workpiece is sustained by the viscous, unpolymerized resin. Highly viscous resins can be used to reduce the velocity gradient, prevent gravitational sedimentation and curb inhibitor diffusion. The well-established mathematical framework for CT can be adapted to optimize TVP implementations. Orth et al.^19^ developed methods to systematically correct for non-parallel beam in TVP, including non-telecentricity and container lensing. Madrid-Wolff et al.^20^ alleviated the diffusive blurring in scattering resins by compensating for the inherent low-pass filtering as per the depth of light penetration.

The introduction of innovative apparatus designs^21^ and resin categories^22–24^ also expands the applicability of TVP. Table 1 compiles the resin recipes previously explored for TVP. In TVP, the desired incident dose in one particular voxel is achieved by exposing it from different directions and thus voxels that are not intended to be cured will still receive a low dose of light. The ideal curing profile is, therefore, one characterized by an induction threshold below which nothing happens, followed by a strong rise in the degree of curing as a function of dose. The free radical photopolymerization of acrylate is close to this ideal with an induction dose defined by dissolved oxygen, which serves as a free radical inhibitor, followed by fast polymerization of the acrylate functionality. A very sharp transition out of the induction, however, can make a workpiece susceptible to overexposure (i.e., voxels that are supposed to remain fluidic polymerize due to undesirable irradiation). Also, if resin turbidity increases after crosslinking, the cured portion of a workpiece may scatter doses allocated for voxels that it shadows in the light path, especially during the final rotation of a printing routine. Thiol-ene based resins^22^ show the more tapered transition in response to incident doses and offer greater control over monomer conversion and gelation. These resins also demonstrate highly tunable mechanical responses, whereas acrylate-based resins offer little to no tunability. However, the induction threshold associated with thiol-ene photoresins can be affected by irradiance. This extra means to manipulate gelation may allow more delicate control over workpiece geometry, but it also complicates process upscaling because of the additional constraints on the operating power of light sources.

**Table 1:** Photoresins investigated in tomographic volumetric 3D printing.

Irradiation	Monomers	Photoinitiator and co-initiator	Refs
405 nm	Gelatin methacrylate in phosphate buffered saline	Lithium phenyl(2,4,6-trimethylbenzoyl) phosphinate	^17^
405 nm	Triethylene glycol diacrylate	2-Methyl-4′-(methylthio)-2-morpholinopropiophenone	^22^
405 nm	Triethylene glycol diacrylate and tris[2-(acryloyloxy)ethyl] isocyanurate	2-Methyl-4′-(methylthio)-2-morpholinopropiophenone	^22^
405 nm	Tri-allyl isocyanurate and tris[2-(3-mercaptopropionyloxy)ethyl] isocyanurate	2-Methyl-4′-(methylthio)-2-morpholinopropiophenone	^22^
405 nm	Triethylene glycol diallyl ether and tris[2-(3-mercaptopropionyloxy)ethyl] isocyanurate	2-Methyl-4′-(methylthio)-2-morpholinopropiophenone	^22^
405 nm	Di-pentaerythritol pentaacrylate	Phenylbis(2,4,6-trimethylbenzoyl) phosphine oxide	^18,20^
405 nm / 455 nm	Bisphenol A glycerolate (1 glycerol/phenol) diacrylate and poly(ethylene glycol) diacrylate	Camphorquinone and ethyl 4-dimenthylamino benzoate	^16,19^
Green	Gelatin methacrylate in phosphate buffered saline	Tris(2,2-bipyridyl) dichlororuthenium(II) hexahydrate and sodium persulfate	^16^
405 nm	Polysiloxane substituted precursor and 1,4-butandiol diacrylate	Diphenyl (2,4,6- trimethylbenzoyl) phosphin oxide	^23^
442 nm	Trimethylolpropane ethoxylate triacrylate and hydroxyethylmethacrylate and silica glass nanocomposite resin	Camphorquinone and ethyl 4-dimenthylamino benzoate (w/ 2,2,6,6-tetramethyl-1-piperidinyloxy as inhibitor)	^24^

Introducing to TVP a mechanism that controls the internal property variation of a workpiece may further expand its field of applications. One possibility is to selectively cure a mixed acrylate-epoxy monomer solution. This idea of separately polymerizing epoxy and acrylate monomers was pursued by Decker^25^, who used a source above 350 nm to crosslink the acrylates, followed by the curing of epoxy with light below 350 nm. Ruiter et al.^26^ implemented a dual-cure process on a synthesized compound with both acrylate and oxetane functional groups. The free radical polymerization was triggered by using filtered light above 385 nm and the cationic polymerization below 385 nm. Larsen et al.^27^ succeeded in using widely separated wavelengths (365 nm and 455 nm) to initiate the orthogonal crosslinking mechanisms. The single resin can thus be cured, using unfiltered light from commercially available LEDs, into a range of multimaterial structures from soft hydrogel to hard solid. The use of acrylate-epoxy chemistry in a conventional multicolor vat photopolymerization setup was investigated by Schwartz and Boydston^11^. The authors used visible light and 365 nm UV to realize spatially controlled microscopic chemical heterogeneity and macroscopic mechanical anisotropy. Another inspiring result is the solution mask liquid lithography^28,29^. Dolinski et al. used photochromic dyes to generate a multicolor curing plane that recedes across the curing volume. This ingenious light confinement resembles that of the xolography^14^ but sidestepped the need for mechanically moving parts. The authors further demonstrated that the interfacial structure between multimaterial regions inherited the strength of the stronger material.

Here we show that a mixed solution of acrylate and epoxy monomers can be cured in a dual-color TVP setup to realize high precision control of internal mechanical properties. TVP excels at delivering predefined light doses to specific points in 3D. When operating in multicolor mode, TVP specifies the dose ratio between different wavelengths for each voxel, which translates to voxel-by-voxel chemical and mechanical property-customization when the aforementioned orthogonal polymerization is employed. Introducing an epoxy monomer to an acrylate resin also changes the nonlinearity of its photoresponse without affecting the curing threshold set by the radical inhibitor concentration, offering an extra tuneability for improving the geometric fidelity of TVP printouts.

## Results

### Dual-color tomographic volumetric 3D printing (DCTVP)

When a resin that responds differently to two different wavelengths is used objects with graded stiffness can be produced. This requires the specification of the dose ratio between wavelengths for each voxel individually, which was realized in our setup by installing two light sources that function in parallel in a TVP setting (Supplementary Fig. 1). As resin we chose to use a mixture of bisphenol A glycerolate diacrylate (BPAGDA), poly(ethylene glycol) diacrylate (PEGDA), and 3,4-epoxycyclohexylmethyl 3,4-epoxycyclohexanecarboxylate (EEC) monomers^27^. Because acrylates polymerize via the free radical mechanism whereas epoxy follows a cationic polymerization mechanism, this orthogonal chemistry enables us to initiate polymerization selectively and create a functionally graded material. In detail this means that visible light excites the free radical photoinitiator (PI) and kicks off the polymerization of acrylates without initiating the epoxy group. UV irradiation causes the cationic PI to produce a Brønsted acid upon excitation and initiates the polymerization of epoxy monomers. The cationic PI also triggers the production of free radical species and initiates the acrylate monomers. Two interlaced polymer networks that are not covalently linked thus form through two independent mechanisms, offering highly tunable mechanical properties.

In DCTVP, an object can be designed so that its internal parts have varied properties (Supplementary Fig. 2a). Each property grade corresponds to a pre-determined dose ratio between blue (~455 nm, source 1) and UV (~365 nm, source 2) lights. The projection sequence for each source was computed separately from the greyscale images reflecting these ratios (Supplementary Fig. 2b). In comparison, single-color TVP uses binary images in which the solid pixels correspond to the curing dose of the chemicals listed in Table 1. We observed that neither naïve nor Ram-Lak filtered back-projection produced a satisfactory combination of brightness and contrast in simulated dose build-up. We thus used a polymerization simulator in an iterative forward-projecting approach with thresholding to generate corrections for sinograms (Supplementary Fig. 3). The two light sources may operate in parallel or in series. Supplementary Figure 2c shows example patterns from two sequences that can be synchronized to print an object with graded internal stiffness in one shot.

### Generating internal stiffness gradients

**Fig. 1:**
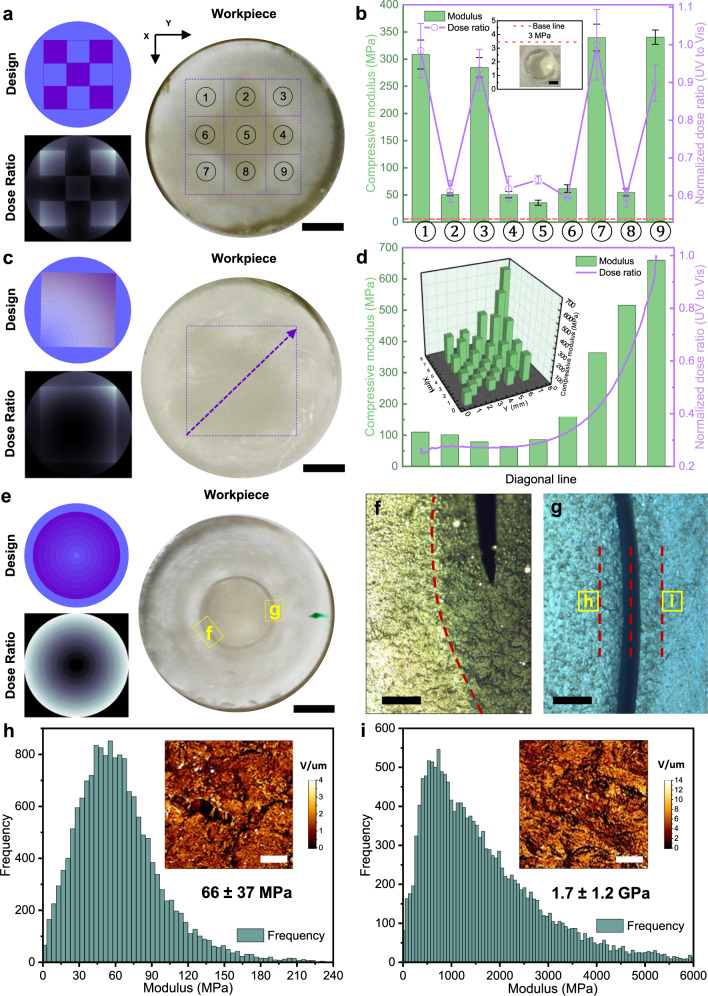
Coordination of two light sources in tomographic volumetric 3D printing grants a high degree of freedom to customize the spatial variation of mechanical properties inside a workpiece. **a** The design, theoretical dose ratio (UV to visible light) and a photo of a 3 × 3 binary grid composite workpiece. The colors in the design pattern indicate the anticipated build-up of light doses of different wavelengths in the X–Y plane (parallel to the incident beams): blue—visible light; purple—UV light. The theoretical dose ratio was calculated using the inhibitor diffusion model. **b** Compressive modulus (green columns) of the nine zones (①–⑨). The mean values of the simulated dose ratio (purple line), averaged over each zone, is plotted on the right axis. The, respectively, colored error bars provide the standard deviation in the measured compressive modulus and dose ratio. Inset: the same geometry printed in single-color mode using visible light. **c** The design, theoretical dose ratio, and a photo of a gray sheet composite structure. **d** Compressive modulus along the diagonal (indicated by the purple arrow in the photo). Inset: mapping of surface stiffness over the entire square area (27 measurements). **e** Design, theoretical dose ratio and a photo of a radially graded composite sample. A circular crack appeared spontaneously. The two marked zones were further studied. **f** Bright-field optical image of the area f of the sample. A sharp contrast in sample transparency was observed 150 µm outside the circular crack (red dashed line, photo taken using a Nikon eclipse LV100ND optical microscope). **g** Zones of interest were identified on both sides of the crack. The spacing between the dashed lines is 150 µm (observed with the AFM embed camera). **h**, **i** Histograms of AFM nanoindentation measurements of zones **h** and **i**. Insets: 25 × 25 µm^2^ a map of stiffness variation in the zone. V/μm measures the slope of the approach force curve and scales with local stiffness. Scalebars: **a**–**c**, **e**: 3 mm; **f**, **g**: 150 µm; **h**, **i**: 5 µm.

Figure 1 shows that internal stiffness gradients could be effectively created using the wavelength-sensitive resin in DCTVP. Figure 1a shows the design, theoretical dose ratio and a photo of a binary 3 × 3 grid printout. In the X–Y plane (parallel to the incident beams, Supplementary Fig. 1), blue light built up uniformly as a cylinder while UV selectively hardened five of the nine zones. Resin AE-3-7 (30% PEGDA/BPAGDA + 70% EEC, see Methods) was first irradiated using the blue and UV patterns simultaneously for 900 s, then the UV sequence alone for another 324 s. Overall the exposure corresponds to an estimate of 10 J and 52 J of visible and UV doses, respectively (Supplementary Table 1 compiles estimated doses for all workpieces and Supplementary Note 1 explains the calculation with an example). This exposure time is longer than the initial demonstrations of tomographic printing^16,17^ because of the diluting effect of epoxy monomers and the slower rate of cationic polymerization compared to free radical polymerization. Simulations suggested that the curing of epoxy at a large radial distance has significantly deterred the delivery of UV dose to the central square (Zone ⑤) because of the increased turbidity of the polymerized resin. This spatial variation of turbidity corresponded well with the simulated dose ratio and could be visually identified in the photo. We expect that the light scattering associated with this heterogeneity is avoidable if a single component resin was employed, where internal property gradient generation relies on greyscale irradiation without forming micro-domains of phase-separated materials^30^.

We measured the compressive modulus of the nine zones as a measure of their stiffness using a ∅1 mm probe of a texture analyzer (TA). Zones ②, ④, ⑥, and ⑧ were primarily cured by blue light and demonstrated an average modulus of ~50 MPa (Fig. 1b), significantly softer than zones ①, ③, ⑦, and ⑨ (~300 MPa on average). This large contrast in stiffness was realized within 3 mm, which corresponded to a modulus gradient ≥80 MPa/mm. Figure 1b also shows the simulated dose ratio for the nine zones. The spatial variation in measured modulus corresponded directly to this ratio (averaged over each zone), with zone ⑤ being an outlier. This zone showed the lowest modulus among the nine, lower even than the four cured primarily using blue light. Visual inspection also suggested that the transparency of zone ⑤ resembles that of crosslinked acrylate rather than epoxy. We speculate that our model did not capture this exception because it did not consider the absorption and scattering by the cured resin outside the simulation domain (the actual curing volume was slightly larger than the domain, the diameter of which equaled the diagonal of the grid). The softer zones in the 3 × 3 matrix were nonetheless significantly stiffer than the same resin cured using single-color TVP. We printed the same geometry using only blue light for 900 s and the same measurement yielded a compressive modulus of 3 MPa without postprocessing (Fig. 1b inset). We attributed this difference in stiffness to the UV dose received inevitably by the softer zones. The iterative sinogram generation algorithm employed in this study computed pattern sequences that guarantee only limited dose contrast in reconstruction. For example, the shape of dose build-up when printing a square post function resembles a normal distribution rather than a step function (Supplementary Fig. 6). In addition, the achievable contrast in dose build-up depends on the heuristic thresholding in pattern generation and can be limited without the nonlinear response of the polymer resin (and the polymerization of epoxy does not possess this nonlinearity). In this case, the limited contrast led to undesired UV dose build-up in the softer zones. We present a quantitative treatment of this subject in Supplementary Notes 2–4 with Supplementary Figs. 4–7.

To demonstrate the capacity of making a functionally graded continuous material with a large variation in elastic properties, we printed the gray sheet design shown in Fig. 1c. The blue pattern remained uniform, whereas the UV pattern increased continuously in intensity from the lower left to the upper right. The object was also printed by irradiating the AE-3-7 resin using blue and UV sequences simultaneously for 900 s then UV alone for another 324 s (Supplementary Table 1). Compressive modulus measurements again verified that the internal stiffness gradient could be controlled by leveraging dose ratio (Fig. 1d). Nine measurements along the primary diagonal showed that the local stiffness correlated positively with the imbued UV dose. The modulus increased continuously from 50 to 650 MPa within 5 mm, corresponding to an average gradient of 120 MPa/mm. Moreover, the stiffness mapping of the entire sample surface indicated a good agreement between theoretical dose ratio distribution and the measured properties. Symmetrical positions on both sides of the primary diagonal were tested, and the results were summarized in a 3D histogram (Fig. 1d). The mapping demonstrated the symmetrical character regarding this diagonal, while along the other diagonal direction, a similar upward trend from the center to the corners was observed as predicted. We also analyzed a replicate of this sample using atomic force microscope (AFM)-based nanoindentation and obtained a highly consistent distribution (Supplementary Fig. 8 and Supplementary Table 2). Close visual inspection revealed the turbidity variation which correlated with the nature of the polymer(s). We noted that the center of the sample was slightly softer than its peripheral areas, which we attribute to scattering stemming from micro-domains of phase-separated materials as discussed above^30^.

The width of the interface between areas with different predefined stiffness is an indicator of the spatial resolution with which stiffness can be controlled in DCTVP. We tested the radial resolution of stiffness control by curing a series of concentric circles in the X–Y plane (Fig. 1e). The blue light was used to cure a uniform cylinder while each circle on top corresponded to a unique UV dose build-up and the sample hardens in the radial direction inner out. The exposure times remained the same (vis + UV for 900 s then UV alone for another 324 s). We observed an almost perfectly circular crack appear shortly after the rinsed workpiece was wrapped in an aluminum foil and stored in a dark environment. The crack had a diameter of ~5 mm, corresponding to a position between the 4th and the 5th inner circles of the design. We speculated that the cracking stemmed from the inhomogeneity of the sample’s internal mechanical properties and the thus-caused differential shrinkage between different regions of the material. Therefore, we looked for areas with high stiffness contrast across the crack in the radial direction. Visual inspection using a ×20 mag. the optical microscope revealed that a slightly larger circle marking a sharp contrast in sample transparency was found 150 μm outside the crack (Fig. 1f).

We performed AFM scanning on selected areas near the crack and identified significant mechanical property contrast across the circle. Figure 1g shows an example of a pair-scanning (captured by an integrated camera of the AFM). Areas *h* and *i* were located on opposite sides of the marked boundary, 150 μm away from the crack. Each area was 25 × 25 μm^2^ and nanoindentation measurements were performed by dividing each area into a 128 × 128 matrix and collecting nanoindentation force curve in each zone (~200 × 200 nm^2^) individually. Figure 1h, i shows the histogram of thus collected moduli. The inset is a pseudo-colored map representing the surface stiffness variation of the scanned area. Modulus of area *h* averaged at 66 MPa whereas that of area *i* at 1.7 GPa, marking a transition between consecutive grades within 300 μm. The result suggests that the resolution of stiffness control in the radial direction is 300 μm or better, which translates to an achievable modulus gradient of 5 MPa/μm.

**Fig. 2:**
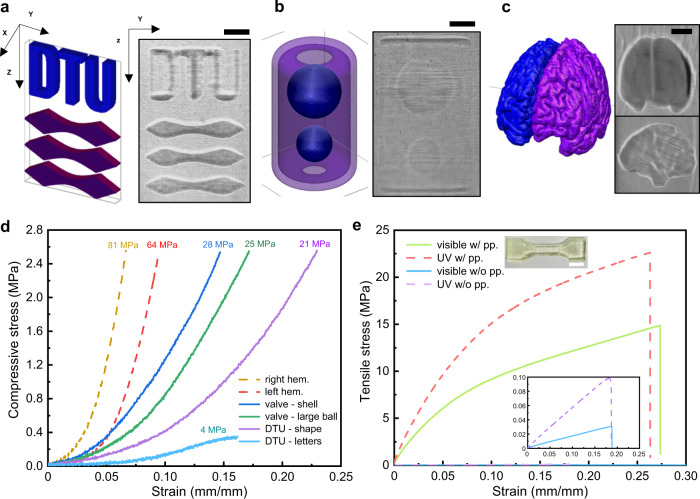
Stiffness can be controlled in workpieces of complex shape and in all three Cartesian directions. **a** Stiffness control in the vertical direction. Left: dual-color design of a DTU logo, in which the letters are cured using visible light and the three stylized lions (shape) below using UV. This is a suspending structure that would require auxiliary support if built using conventional AM methods. Right: A DTU logo printout floating in viscous resin, captured by the surveillance camera. DCTVP can print this multimaterial workpiece in one shot without auxiliary support. **b** Stiffness control in a nested structure. Left: dual-color design of two encaged balls (valve). The hard shell is cured using UV while the soft balls inside using visible light. Right: a snapshot of the polymerized workpiece captured by the surveillance camera. **c** Dual-color design of a 3D brain model (left). The left hemisphere is cured using visible light and the right hemisphere using UV. Two surveillance snapshots of a printout with good geometric fidelity are shown on the right. The front view (top) suggests that the right hemisphere (hem.) was more susceptible to overexposure because the UV source had a greater volumetric printing rate. The gyri features can be seen in the side view (bottom). Scalebars: 3 mm. **d** Stiffness characterization of the workpieces in Fig. 2a–c. It was noted that the modulus extracted from these response curves, obtained using a texture analyzer, were subject to uncertainties associated with the size, shape and the internal homogeneity of the test subjects. For these irregular geometries, it was not possible to prepare standard-shaped test specimens as we did for the samples in Fig. 1. Therefore, the numbers reported here reflect only the relative stiffness of various parts in a single printout and are meant for intrasample comparison only. **e** Standard tensile test results. The dogbone specimens were printed using each of the two light sources individually. The results for specimens without postprocessing (pp.) are shown in the inset. Scalebar: 5 mm. The dimensions of the specimen are given in Supplementary Fig. 9.

Stiffness can also be controlled in workpieces with more complex geometries and along all Cartesian axes (Fig. 2). An advantage of TVP is to print suspended structures without auxiliary support. We tested stiffness control in such a structure by printing a DTU logo using resin formula AE-3-1 (Fig. 1e). The three letters were cured using blue light for 708 s and the three stylized lions below using UV in parallel for 510 s. TA measurement indicated that the UV-cured part was over four times stiffer than the letters, confirming that the mechanical property can be controlled in the Z direction. It was noted that the modulus extracted from the TA responses were affected by the size, shape, and homogeneity of the test subjects and were presented in Fig. 2d for intrasample comparison only. Another advantage of TVP is to print nested structures in one shot. We designed the encaged balls (Fig. 2b) to show that stiffness can be controlled in both radial and axial directions for a nested structure with suspended parts. Two soft, floating balls of varying diameters were sheltered by a hard shell with small openings on both ends. This axially symmetric structure was printed by first irradiating the UV pattern (shell) for 180 s then switching on the blue pattern (balls) and allowing both sources to work in parallel for another 360 s. TA analysis showed that the shell thus obtained was ~12% stiffer than the larger ball.

We then investigated the achievable stiffness contrast using DCTVP by printing two hemispheres of a brain model using different light sources (Fig. 2c). The sinograms were computed in such a way that the left hemisphere would be cured exclusively by blue light whereas the right hemisphere would be cured by UV. In contrast to the DTU logo, in which the two projectors operated at different heights and thus did not interfere with each other, the hemispheres had the same elevation and inevitably received undesirable irradiation from the other light source. This contamination effectively reduced the achievable stiffness contrast. When printing, the visible pattern sequence was projected on the curing volume for 54 s, after which the UV sequence was switched on and the two sources operated in parallel for 300 s. The UV was then turned off and the visible light operated alone for another 52 s. The overall exposure time was 300 s for UV and 406 s for visible light. This exposure sequence was the result of extensive trial-and-error to optimize the geometric fidelity of the printout. The feedback was provided by judicious analysis of the printing process recorded by the surveillance system and with the aid of the inhibitor diffusion model. The right hemisphere thus printed was ~26% stiffer than the left hemisphere. As a reference, we also printed dogbone specimens for standard tensile tests (Supplementary Fig. 9) using the two light sources individually. These specimens received no undesired irradiation and thus represented the maximum achievable stiffness contrast for the given geometry. Tensile testing showed that, without postprocessing, the specimen printed using UV was ~241% stiffer than the one printed using blue light (*E* = 542 kPa vs. 159 kPa, Fig. 2e). After postprocessing, both specimens hardened significantly (201 MPa vs. 127 MPa), and the stiffness contrast reduced to 58%. Fourier-Transform Infrared Spectroscopy (FTIR) analysis showed two signature peaks of the epoxy group near 1430 and 790/cm in the visible light-cured specimen without postprocessing (Supplementary Fig. 10). We attribute the sample hardening after postprocessing to the crosslinking of residual functionalities.

### Geometric fidelity vs. Stiffness contrast

In this section, we use model simulations to show that a balance needs to be stricken between the geometric fidelity and the achievable property contrast. In DCTVP, the geometric fidelity of a workpiece is determined by the spatiotemporal evolution of radical inhibitor distribution in the curing volume. In this study, both oxygen from the atmosphere and the 4-methoxyphenol (100 ppm) shipped with the acrylate monomers contributed to radical inhibition. The initial concentration of effective inhibitors was a constant for each monomer batch but might vary slightly between batches. In modelling, we did not differentiate between the inhibitors and assumed a constant and normalized initial inhibitor concentration. Inhibitor transport is determined by the diffusive Damköhler number

1 $$Da=\frac{{\tau }_{{{{{{\mathrm{diff}}}}}}}}{{\tau }_{rxn}},$$

in which *τ*_*diff*_ is the characteristic time of inhibitor diffusion (s)

2 $${\tau }_{{{{{{\mathrm{diff}}}}}}}=\frac{{l}^{2}}{{D}_{A}}.$$

**Fig. 3:**
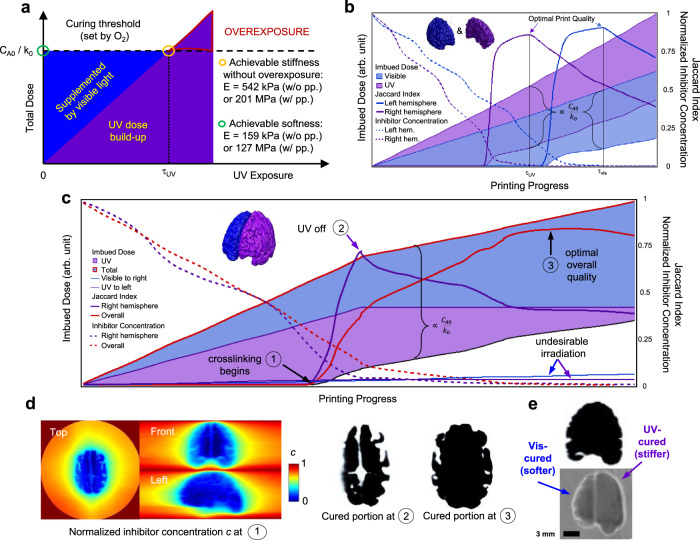
The achievable stiffness contrast in DCTVP is limited by the curing threshold of free radical polymerization set by the initial inhibitor concentration. **a** Both light sources contribute to free radical polymerization. The dual-color design of a multimaterial object starts with the design of the anticipated UV dose distribution (purple shaded), which spatially defines the relative stiffness inside the workpiece. The visible light dose build-up is then calculated to supplement doses to voxels that would not receive sufficient UV irradiation to initiate the crosslinking (blue shaded). The softest workpiece (green circle) would be produced when the curing dose is supplied only by the visible light source. Similarly, the hardest workpiece (yellow circle) would be produced if it received only UV irradiation. *τ*_*UV*_ is the exposure time when an object was cured using UV alone. Beyond *τ*_*UV*_, the object keeps hardening, but its geometric fidelity decreases because the voxels in its vicinity polymerize undesirably. **b** Evolution of imbued doses, printing quality (measured using the Jaccard index), and normalized inhibitor concentration in the targeted subvolume if the two hemispheres of the brain model were printed individually using single-color TVP. Only doses contributed to inhibitor consumption are shaded. The blank space below the shaded areas indicates the doses absorbed by cured resin. **c** Evolution of imbued doses, printing quality, and normalized inhibitor concentration when the two hemispheres were printed simultaneously using DCTVP. The two thin solid lines show the UV irradiation received by the left hemisphere (purple) and the visible light irradiation received by the right hemisphere (blue). **d** Distribution of inhibitor concentration (*c*) in the curing volume at time point ① (polymerization initiation) and the cured portions (top view) at time points ② (optimal UV exposure) and ③ (optimal overall quality). **e** The front view of the cured portion when the curing sequence in **c** was followed. Top: model simulation; bottom: surveillance snapshot.

*l* is the voxel size (m) and *D*_*A*_ the effective diffusivity of inhibitor in the resin (m^2^/s). *τ*_*rxn*_ is the characteristic time of photo-induced inhibitor consumption and is determined by the energy balance:

3 $${C}_{A0}V={k}_{0}{\int }_{\!\!\!{0}}^{{\tau }_{rxn}}{\int }_{\!\!\!\!{V}}{\int }_{\!\!\!{\lambda }}{\alpha }_{\lambda }{I}_{\lambda }d\lambda d{{{{{\bf{r}}}}}}dt$$

in which *C*_*A0*_ is the initial concentration of effective inhibitors (mol/L), *V* is the volume of the resin that would receive irradiation during rotation (m^3^), *k*_*0*_ is inhibitor consumption per unit energy (mol/J), *λ* is the wavelength of incident light (nm), *α* is absorption coefficient (m^−1^) and *I* the differential radiant flux per spectrum (mW·m^−2^·nm^−1^). Equation (3) also gives the volumetric printing rate (m^3^/s) of TVP:

4 $${Q}_{v}=\frac{V}{{\tau }_{rxn}}=\frac{{k}_{0}}{{C}_{A0}}{\int }_{\!\!\!\!{V}}{\int }_{\!\!\!{\lambda}}{\alpha }_{\lambda }{I}_{\lambda }d\lambda d{{{{{\bf{r}}}}}}\propto \frac{{k}_{0}{I}_{0}}{{C}_{A0}},$$

in which *I*_0_ is the output power of the light source (mW). It is desirable to operate TVP at high *Da* so that diffusion does not smear out small features. However, shortening *τ*_*rxn*_ makes the workpiece more susceptible to overexposure when printing quality is controlled by visual inspection. For any given voxel P in the curing volume, its curing threshold (total light dose imbued before free radical polymerization takes place) is set by the concentration of pre-dissolved inhibitors (*C*_*A0*_/*k*_*0*_, Fig. 3a) while its stiffness is controlled by the build-up of UV dose. Because UV also contributes to the initiation of free radical polymerization, the total amount of UV dose allowable—and thus the maximum stiffness achievable before voxels surrounding P polymerize undesirably—is also determined by *C*_*A0*_. Standard tensile tests showed that, with this extra constraint, the softest material printed in this study had a Young’s modulus of 159 kPa (green circle in Fig. 3a) and the hardest, 542 kPa (yellow circle) before postprocessing. In designing dual-color patterns, the UV sequence was first computed according to a desirable internal stiffness gradient, after which the visible patterns were generated to supplement the required dose to reach the curing threshold.

Figure 3b shows the simulated evolution of key parameters as exposure time increases when the two hemispheres of the brain model were printed individually using single-color TVP. The output power of both sources and the attenuation coefficient of the curing volume was adjusted according to the resin response data (Supplementary Fig. 11) and the experimental trial-and-error results. The shaded areas indicate the light doses that contributed to the initiation of free radical polymerization. They are upper-bounded by the total irradiation received by the curing volume and lower-bounded by the doses absorbed by polymerized resin. The area scales with *C*_*A0*_/*k*_*0*_ and changes gradually over time because of the increased turbidity and thus light attenuation after resin polymerization (Eq. (3)). Jaccard index (*J*), the extent of overlap between design and printout, was used as a measure of print quality. The ideal exposure time (τ_opt_) was determined at the maxima of *J* and the period of projecting pattern sequence was adjusted so that τ_opt_ equals an integer number of the rotation periods. In this study, τ_opt_ for the left (τ_vis_) and the right (τ_uv_) hemispheres were experimentally determined to be 690 and 330 s, respectively. The dashed lines show the depletion of inhibitors in the targeted subvolume. In both cases, local polymerization took place before the complete depletion of inhibitors and overexposure occurred immediately after the subvolume became inhibitor-free. This sensitivity to inhibitor distribution poses a significant challenge for geometric fidelity assurance when operating TVP at large Damköhler numbers.

Figure 3c shows the evolution of key parameters when two light sources were coordinated to print the hemispheres in parallel. The shape of the printout was determined by inhibitor depletion induced by both sources. The concentration gradient existed because of the uneven build-up of doses as well as inhibitor diffusion, and local polymerization occurred first near the rotation center (Fig. 3d – ①). In our setup, the UV source offered a greater *Q*_*v*_ and the Jaccard index for the right hemisphere reached a maximum before the left hemisphere was properly cured. We turned off the UV at point ②, after which the curing dose needed to reach the maximum of the overall *J* was supplemented by visible light only. Figure 3d shows the cross-sections along the X–Y plane of the cured geometry at time points ② and ③. The geometric fidelity of the right hemisphere after time point ② was compromised by the undesirable irradiation (contamination) it received from the visible light source. The simulation suggested that the contamination was only a very small portion of the overall imbued dose (Fig. 3c shows undesirable irradiation received by the two hemispheres separately). However, because the local inhibitor concentration remained very low at the vicinity of the optimally cured geometry, the resin became very sensitive to excessive light dose. As a result, the UV-cured part was particularly susceptible to overexposure even though the source was shut down at an optimal exposure time. In Fig. 3e, we show that the extent of overexposure predicted by the inhibitor diffusion model was in good agreement with this printed geometry captured using the surveillance camera. The impact of the undesirable irradiation could also be inferred from the optimized curing times. Compared to the single-color mode, to reach a similar geometric fidelity in the dual-color mode, the experimentally optimized curing time for the left hemisphere was shortened by 41%, from 690 to 406 s, whereas for the right hemisphere the time was shortened by 9% from 330 to 300 s. This shortening also compromised the achievable stiffness range. If the curing order in Fig. 3c were followed (UV + vis then vis only), the left hemisphere would be stiffer than its counterpart that which was cured in the single-color mode (Fig. 3b) because of UV contamination. It may be tempting to rearrange the curing order (e.g., vis then UV + vis) so that the curing of both hemispheres completed at the same time to achieve maximal fidelity. However, doing so will not only harden the left hemisphere but also reduce the achievable stiffness of the right hemisphere because of the fidelity constraint explained in Fig. 3a. In this study, we achieved a reasonable geometric fidelity through extensive trial-and-error and the compressive modulus of the two hemispheres thus obtained were 81 MPa (right) and 64 MPa (left). Overall, striking a balance between print quality and stiffness contrast is not facile, and understanding the spatiotemporal evolution of inhibitor distribution is non-trivial for improving the efficiency of experimental trial-and-error.

## Discussion

We showed that a workpiece with an internal gradient of mechanical properties can be generated using DCTVP. However, the achievable stiffness contrast can be limited if the detrimental effect of overexposure becomes concerning. Currently, print quality is evaluated experimentally by visual inspection and numerically using Jaccard index. Both introduce uncertainties because the former is qualitative and lacks strict reproducibility while the latter is not always efficient in gauging the fidelity of fine features. Iterative sinogram computation has proven useful in improving printing quality given a good feedback loop^17^. It is particularly tempting to offer such feedback in real-time by exploiting the surveillance system. For example, inhibitor-sensitive fluorescent nanoparticles can be embedded in the resin to reveal the spatiotemporal evolution of inhibitor distribution in the curing volume, which can be reconstructed in 3D directly from images captured using the surveillance camera. Extra leverage against overexposure may be sought by introducing tuneability to the nonlinear response of photoresin to irradiation. An even more attractive option would be developing a strategy that switches certain polymerization mechanisms on or off via external stimulus. If, for example, the free radical polymerization can be switched off at τ_opt_, the printing and the stiffness control will become orthogonal, significantly widening the achievable property range. A similar strategy can be employed to confine workpiece consolidation to only the focal plane of a projector, which will reduce the deleterious effect of beam étendue on printing resolution.

## Methods

### DCTVP assembly

Supplementary Figure 1 shows a schematic design of the apparatus used in this study. The UV and visible light paths were setup orthogonally, nesting the surveillance system in the latter. An off-the-shelf DLP projector (Acer XD1270D) was used with an optical lens (f = 200 mm) to focus the projection into the curing volume, in the same plane as the rotation center. The UV source was provided by a DMD projector (VISITECH-LRS-4KA, Visitech, Norway) and an aspherical lens (f = 200 mm) was installed in front of the beam outlet. The two projecting centerlines were aligned so that they intersected at the rotation axis of the curing volume. The maximal projection area was 36 × 16 mm^2^. The surveillance was illuminated by a collimated LED light source (*λ* = 625 nm, M625L4-C4, Thorlabs). Printing processes (e.g., Supplementary Movies 1–3), which provided feedback to experimental trial-and-error, were recorded using a CCD camera (Grasshopper3 GS3-U3-28S5C, Point Grey Research). A cylindrical test tube 15.5 mm in diameter containing photoresin was mounted to a motorized rotation stage (PRM1/MZ8, Thorlabs) located at the intersection of the two light paths. A cuboid vat containing index-matching fluid was placed outside the test tube. The walls of the vat were perpendicular to the incident beams.

### Sinogram computation

Supplementary Figure 3 shows a flowchart of the iterative sinogram computation routine. The STL files used in this study were retrieved from Thingiverse.com and sliced using ChiTuBox (CBD-Tech, SZX). The gray values of the pixels in the TIFF stack were adjusted in accordance with the target dose distribution. The initial sinogram was computed using naïve forward projection, i.e., for each projecting angle *θ*, the position D(*d*) of the projection of point P(x,y) on a 1D detector was determined by

5 $$d=y\,\cos \,\theta -x\,\sin \,\theta ,$$

and the gray value of P was added to the light intensity at D. The initial sinogram S_0_ thus generated was imported to a print simulator for the evaluation of printout quality. The simulator back-projects the sinogram into a pristine domain primed with a polymer precursor. For each point in the curing volume, the instantaneous energy build-up ($\dot{E}$, joule/s) was

6 $$\dot{E}={I}_{0}\cdot \alpha (L)\cdot \exp \left(-\int _{0}^{L}\alpha (l)\cdot dl\right),$$

in which the dummy variable *l* represents the distance light travelled after entering the curing volume and before hitting the point in question at *l* = *L*. The absorption coefficient *α* evolves spatiotemporally according to the rotation history and the polymer response to incident light. We used a logistic equation to simulate the nonlinear response of free radical polymerization to energy build-up. The critical incident dose was estimated heuristically by applying Eq. (4) to observations made from surveillance recording. The time dependence was simulated by matching the time step *dt* to the projecting frame rate (FPS): *dt* = FPS^−1^. The rotation direction would change both the chirality of the workpiece and the shadowing effect of cured voxels traversed by incident light. The logistic response curve also served as a heuristic thresholding to estimate the shape of the polymerized workpiece from energy build-up. The workpiece was then compared with the original design. For binary geometry, Jaccard similarity coefficient was calculated as:

7 $$J{{{{{\mathrm{(Design,Workpiece)}}}}}}=\frac{{{{{\mathrm{{Design}}}}}}\cap {{{{{\mathrm{Workpiece}}}}}}}{{{{{\mathrm{{Design}}}}}}\cup {{{{{\mathrm{Workpiece}}}}}}}$$

For grayscale images, the Frobenius norm of the difference between the printout and the original design was calculated:

8 $${\Vert {{{{{\mathrm{Difference}}}}}}\Vert }_{F}=\sqrt{\mathop{\sum}\limits_{y}\mathop{\sum}\limits_{x}{|{I}_{x,y}|}^{2}}.$$

in which *I* is the gray value of the pixel designated by (*x*,*y*) after rescaling. A typical loop-exiting criterion was *J* > 0.9 or ${\Vert {{{{{\mathrm{Difference}}}}}}\Vert }_{F}$ did not significantly change after 10 iterations. When not met, the difference was naïvely forward projected to generate the correction for the previous sinogram. The correction may result in negativity, which would be removed (set to zero) before being back-projected in the print simulator. We observed that the negativity removal prevented the sinogram from fully reconstructing the desired geometry. As a result, the heuristic thresholding before the design-workpiece comparison became essential in generating sinograms for satisfactory print quality.

### Diffusion model

The effect of inhibitor diffusion on printing quality was evaluated by solving

9 $$\frac{dc}{dt}={\nabla }^{2}c+Da,$$

in which *c* is the dimensionless inhibitor concentration:

10 $$c=\frac{{C}_{A0}-{C}_{A}}{{C}_{A0}}.$$

*C*_*A*_ and *C*_*A0*_ are the concentration of inhibitor and the initial concentration of inhibitor in the polymer precursors, respectively (mol/L). *Da* is the Damköhler number:

11 $$Da={l}^{2}\frac{{k}_{0}{\int }_{\lambda }\alpha (\lambda )I(\lambda )d\lambda }{{C}_{A0}{D}_{A}},$$

in which *l* is voxel size (m), *D*_*A*_ the diffusivity of inhibitor (m^2^/s), *α* the absorption coefficient and *I* the irradiance (mW) of wavelength *λ* (nm), *k*_*0*_ is the zeroth-order rate constant that relates inhibitor consumption to irradiation. *Da* evolves spatiotemporally as a consequence of changing irradiation angle and nonlinear polymer response. We thus used *Da*_*0*_ as a general indicator of the relative strength between photochemical reaction and inhibitor diffusion:

12 $$D{a}_{0}=\frac{{l}^{2}{k}_{0}{I}_{0}}{{C}_{A0}{D}_{A0}},$$

in which *I*_*0*_ is the output power of projector(s) in mW and *D*_*A0*_ is the initial diffusivity of inhibitor (m^2^/s) before the precursors crosslink. The diffusivity decreases to zero after crosslinking.

Equation (12) was discretized using the same voxelization scheme of the printing simulator in iterative sinogram computation (Supplementary Fig. 12). The Fickian fluxes *N*_*A*_ for each voxel were computed at the six inter-voxel surfaces and the photochemical reaction was treated as a zeroth-order sink ${r}_{A}={k}_{0}{\int }_{\lambda }\alpha (\lambda )I(\lambda )d\lambda$. Inhibitor concentration was assumed uniform inside each voxel. Diffusion was simulated to assist the trial-and-error in determining optimal exposure time to operate TVP in the dual-color mode. Diffusion simulation was not included in the iterative sinogram computation.

### Resin preparation

The wavelength-sensitive resins were prepared by combining acrylate- and epoxy-based photoresins at a volume ratio of 3:7 (AE-3-7) or 3:1 (AE-3-1). The acrylate-based resin was made by mixing bisphenol A glycerolate diacrylate (BPAGDA, CAS# 4687-94-9) and poly(ethylene glycol) diacrylate (PEGDA, CAS# 26570-48-9, average M_n_ 250 w/100 ppm 4-methoxyphenol as inhibitor) at a 3:1 volumetric ratio, with 5 mM camphorquinone (CQ, CAS# 10373-78-1, ≥96.5% purity) and ethyl 4-dimethylaminobenzoate (EDAB, CAS# 10287-53-3) as the photoinitiator (PI) and co-initiator, respectively. The epoxy-based resin was formulated with monomer 3,4-epoxycyclohexylmethyl 3,4-epoxycyclohexanecarboxylate (EEC, CAS# 2386-87-0) and 50 mg/ml cationic initiator triarylsulfonium hexafluoroantimonate salts, mixed (CAT2, CAS# 109037-75-4, 50% in propylene carbonate). The chemicals were obtained from Sigma–Aldrich and used without further purification.

### Resin response

The wavelength-sensitivity of AE-3-7 resin was tested by measuring its responses to incident lights of varied wavelength and intensity (Supplementary Fig. 11). A static image containing 9 small, filled circles of different grayscale values at the rotation center was projected to the curing volume and the reciprocal of the curing time as a function of radiant fluxes of the two wavelengths of light were recorded. The dependence of curing time on incident intensity was thus extracted to aid the experimental trial-and-error in determining optimal exposure for various geometries and to parameterize the inhibitor diffusion model.

### Absorption characterization

A UV-vis spectrophotometer was used to measure the absorption spectra of the monomers before they were mixed to make the wavelength-sensitive resins. The acrylate resin with CQ and EDAB absorbed both the blue light at ~455 nm and the UV at ~365 nm. The epoxy resin with the cationic PI, CAT2, absorbed only the irradiation of the UV at ~365 nm (Supplementary Fig. 13).

### Experimental trial-and-error

To print complex geometries in dual-color mode, it was necessary to iterate on the printing parameters to achieve optimal geometric fidelity. A target geometry would first be cured using each light source individually, which gave the upper limits of the curing times for each wavelength. The curing order, i.e., when to turn on and off the projectors, in parallel or in series, was then determined with the goal to complete the irradiation from both sources at the same time so that the print quality, judging from the surveillance camera, could be maximized.

### Postprocessing

After printing, we used isopropanol to rinse off residual resin attached to the workpiece and left it to dry in a dark environment for 24 h before wrapping it in an aluminum foil. A dark storage environment is essential for preserving the internal property gradients if the workpieces are not purified through solvent exchange^27^. To prepare samples for TA and AFM analysis, cylindrical samples presented in Fig. 1 were first sliced using a miter saw into pieces of 5 mm thickness. The surfaces were then polished using a series of sandpapers (Grit 800 ≥ 1500 ≥ 4000). Additional postprocessing that led to sample hardening (the dogbones for standard tensile tests) was conducted by heating a sample to 60 degree and exposing it to 405 nm UV for 4 h using the *Form Cure* automate postprocessing machine (FH-CU-01, Formlabs).

### Mechanical characterization

Compressive and tensile tests were conducted using a Texture Analyzer (Stable Micro Systems, Godalming, UK). When measuring compressive modulus, a cylindrical metal probe 1 mm in diameter was used with an advancing speed of 0.1 mm/s. For tensile testing, 100% elongation was used as the terminal condition and the traction speed was 0.1 mm/s. The compressive or tensile stress was recorded as a function of strain, from which the corresponding moduli were extracted.

AFM nanoindentation measurements (NanoWizard III, JPK Instruments AG, Berlin, Germany) were conducted using sharp-tip cantilevers (HQ:NSC15/AL BS, with resonance frequency 325 kHz, MikroMasch, USA). The spring constant of the cantilever was estimated using the thermal noise method. Areas of interest were identified with the aid of an integrated optical microscope. For each area, force measurements were conducted using an approaching speed of 0.5 μm/s and a set point of 250 nN over a 5 × 5 μm^2^ area (7 × 7 lattices). The modulus of the samples was estimated using a Hertzian or a DMT model for a conical tip shape. The Hertzian model was chosen when the adhesion between AFM tip and the sample surface was negligible whereas the DMT model was used when the adhesion cannot be ignored. The force curves collected over different parts of the greysheet sample suggested the presence of both cases. AFM imaging was conducted in the Quantitative Imaging (QI) mode over 25 × 25 μm^2^ areas where the surface topography and modulus of the surface were mapped. The QI mode collects approach-retract force curves and extract information regarding relative topographical height, relative stiffness, and adhesion. The insets in Fig. 1h–i are stiffness images. Each pixel is represented by a value corresponding to the slope of the approach force curve in the indentation regime. The slope has a unit of V/μm because we normalize the vertical deflection of the cantilever (characterized by the variation of the laser position reflected from the cantilever and has a unit of volt on the detector) by the indentation depth (μm). The value of the slope scales with the stiffness of the surface.

### Statistical information

The compressive modulus values in Fig. 1b were averaged over 4 measurements using the TA probe at different locations in the same zone of the 3 × 3 binary grid (*n* = 4, error bar shows standard deviation). The mean modulus shown in Fig. 1h was calculated from the force curves (*n* = 16300) taken in the indicated zone and the uncertainty referred to the standard deviation of these measurements. Similarly, *n* = 16128 for Fig. 1i and the standard deviation was reported as a measure of uncertainty.

## Supplementary information

Supplementary Information

Description of Additional Supplementary Files

Supplementary Movie 1

Supplementary Movie 2

Supplementary Movie 3

Supplementary Software 1

## Data Availability

All data generated or analyzed during this study are included in this published article and its supplementary information files. Source data are provided with this paper.

## Source data

Source Data

## Code availability

Exemplary MATLAB scripts for iterative sinogram computation (SC1. Iterative Sinogram Computation.m) and print simulation with inhibitor diffusion (SC2. Print simulation with inhibitor diffusion.m) are provided as supplementary software.
