# Supplementary material for: Stiffness control in dual color tomographic volumetric 3D printing
Source: Nat Commun. 2022 Jan 18;13:367. doi: 10.1038/s41467-022-28013-4 (PMC8766567; doi:10.1038/s41467-022-28013-4)
Supplement: Supplementary file 1 — Supplementary Information [file 41467_2022_28013_MOESM1_ESM.pdf]

Supplementary Information for

Stiffness Control in Dual Color Tomographic Volumetric 3D Printing

Bin Wang[1], Einstom Engay[2], Peter R. Stubbe[3], Saeed Z. Moghaddam[4], Esben Thormann[4], Kristoffer Almdal[4], Aminul Islam[1], Yi Yang[4,5]*

[1] Department of Mechanical Engineering, Technical University of Denmark, 2800 Kongens Lyngby, Denmark

[2] National Center for Nano Fabrication and Characterization, Technical University of Denmark, 2800 Kongens Lyngby, Denmark

[3] National Food Institute, Technical University of Denmark, 2800 Kongens Lyngby, Denmark

[4] Department of Chemistry, Technical University of Denmark, 2800 Kongens Lyngby, Denmark

[5] Center for Energy Resources Engineering, Technical University of Denmark, 2800 Kongens Lyngby, Denmark

* To whom correspondence should be addressed (email: yyan@dtu.dk)

Supplementary Figures 1-13

Supplementary Tables 1-2

Supplementary Notes 1-4

Supplementary Figures 1-13

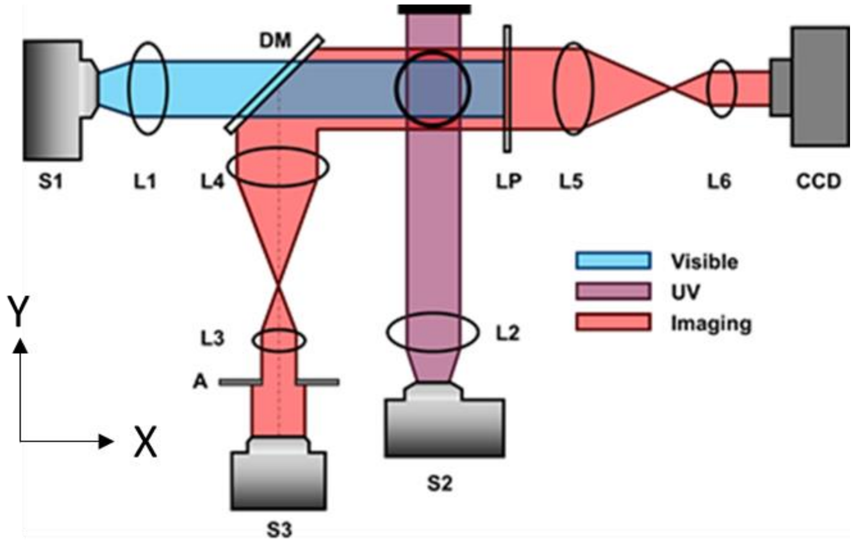

Supplementary Figure 1. Schematic drawing of the dual-color TVP setup. S represents the projectors and the illumination light source; L represents the lenses; DM represents the dichroic mirror (DMSP490L Thorlabs, 490 nm Cutoff), utilized to transmit the visible light with wavelength under 490 nm from S1, and reflect the long-wavelength light from S3. LP represents the long-pass filter (610 nm long pass, FGL 610S, Thorlabs), which was used to block the light from S1 so that the camera will only detect the illumination from S3.

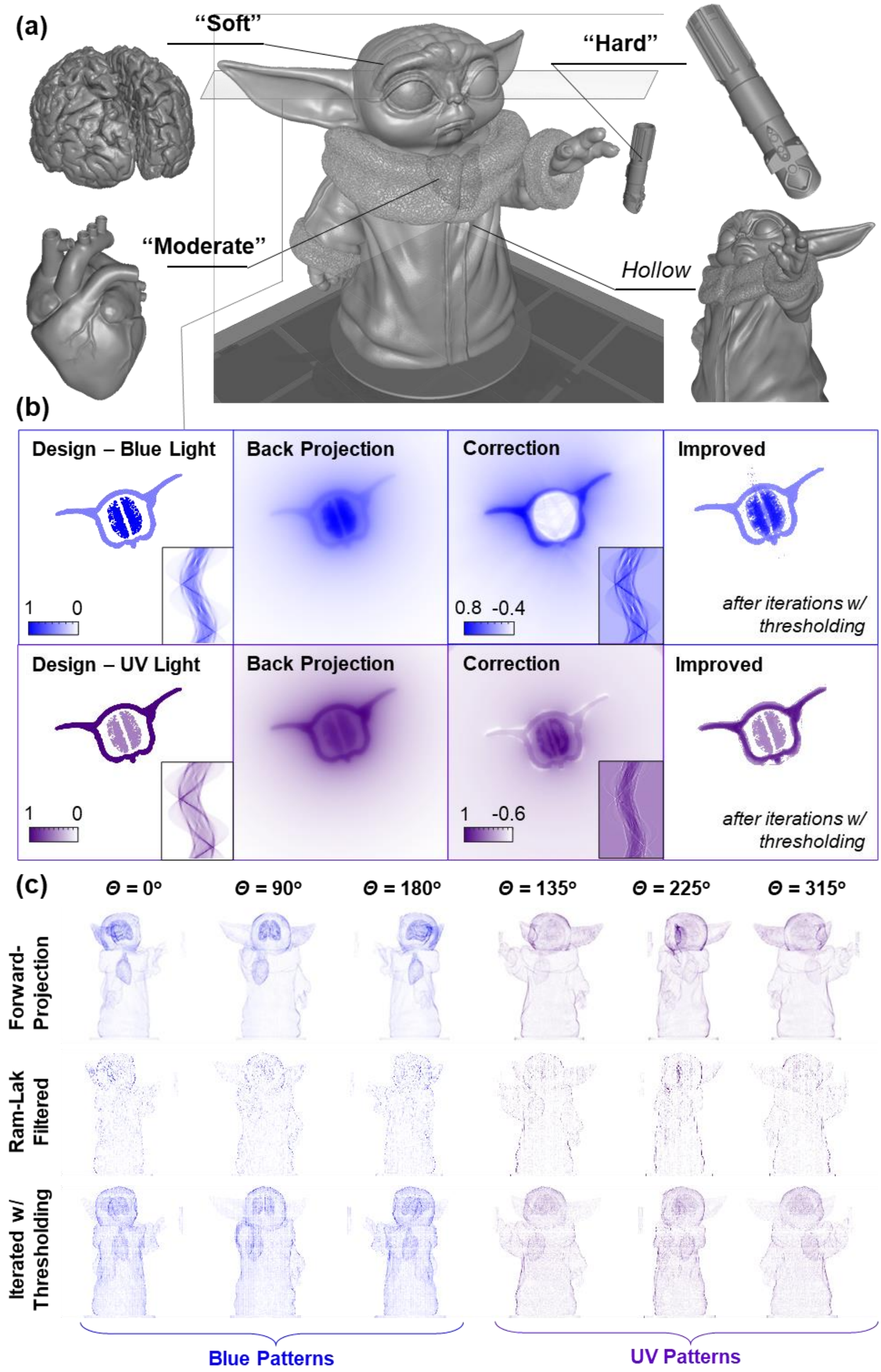

Supplementary Figure 2. An object containing multiple grades of mechanical properties can be 3D printed at a high volumetric rate by using a wavelength-sensitive photoresin

in a TVP setup with two light sources. (a) The solution of acrylate and epoxy monomers can be selectively photopolymerized using blue (λ ~= 455 nm) and UV (λ ~= 365 nm) light to produce materials with varied mechanical properties. An object that contains multiple grades of stiffness can be designed according to a pre-establish correlation between the blue-to-UV dose ratio and said property. TVP also allows the rapid printing of hollow/nested/enclosed structures in one-shot. (b) The sequences of projecting patterns are computed separately for each light source from greyscale images that define the desired spatial distribution of dose build-up in the curing volume. The sinograms can be attained from a direct forward projection of the grayscale image or be corrected iteratively to improve reconstruction fidelity. (c) Examples of projecting patterns for the two light sources. For blue light, the softer parts are "brighter" and for UV vice versa.

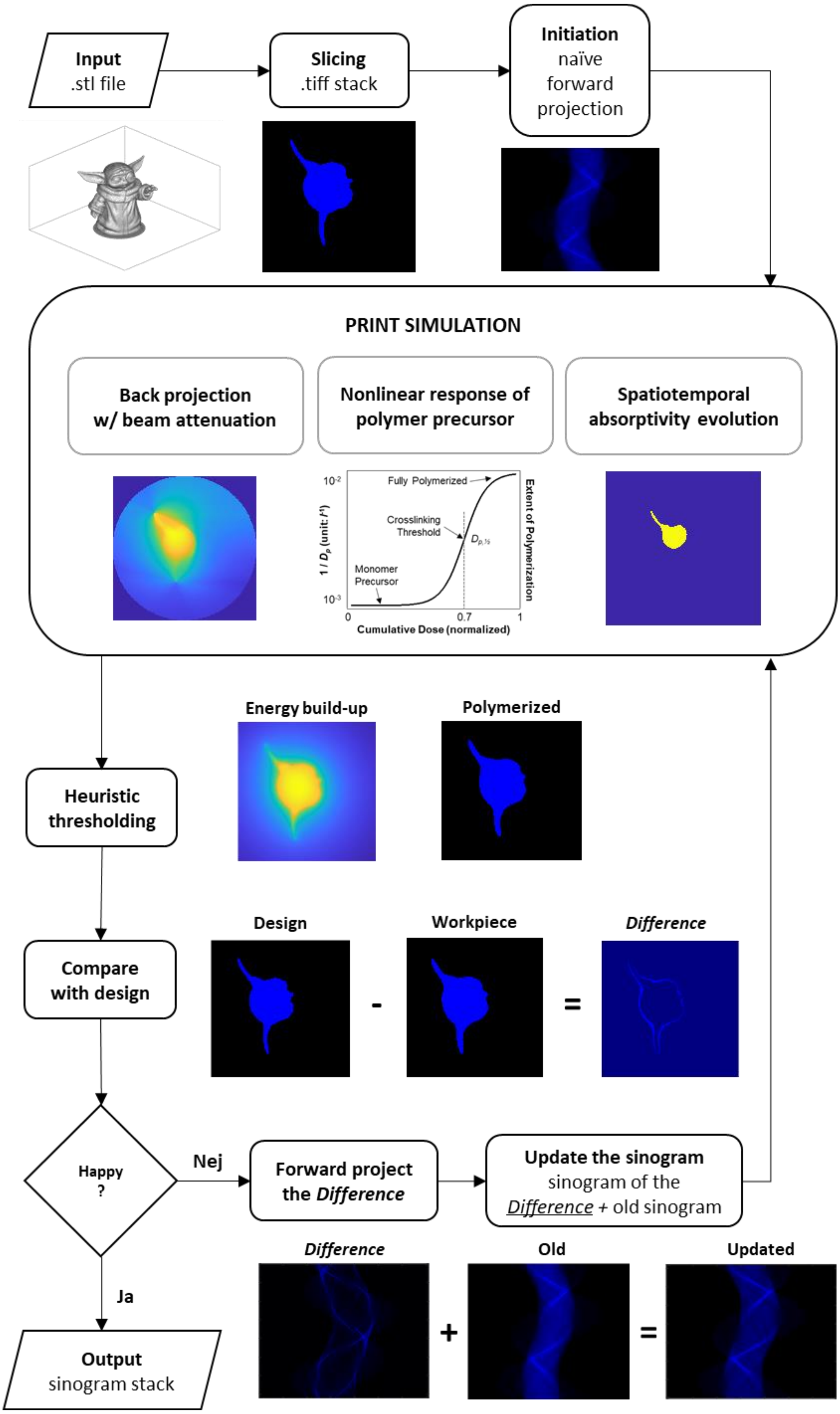

Supplementary Figure 3. Flow chart of the iterative routine for sinogram computation.

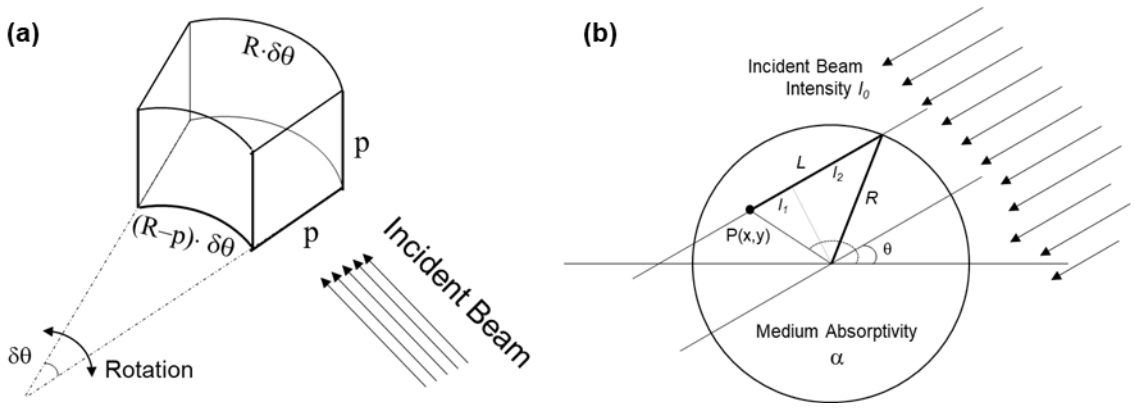

Supplementary Figure 4. The voxel size is determined by projector specifications and differs for each light source. (a) The voxel in TVP takes the shape of a fan-shaped cube and is defined by two characteristic lengths (*p* and $R{\cdot}\delta\theta$) simultaneously. (b) The critical exposure time at point P(*x*,*y*), required to reach the designed dose, is determined by the incident beam intensity, the rotating speed of the curing volume and the medium absorptivity. This critical time further sets a cap for the tangential voxel edge that correlates with the refresh rate of a light source.

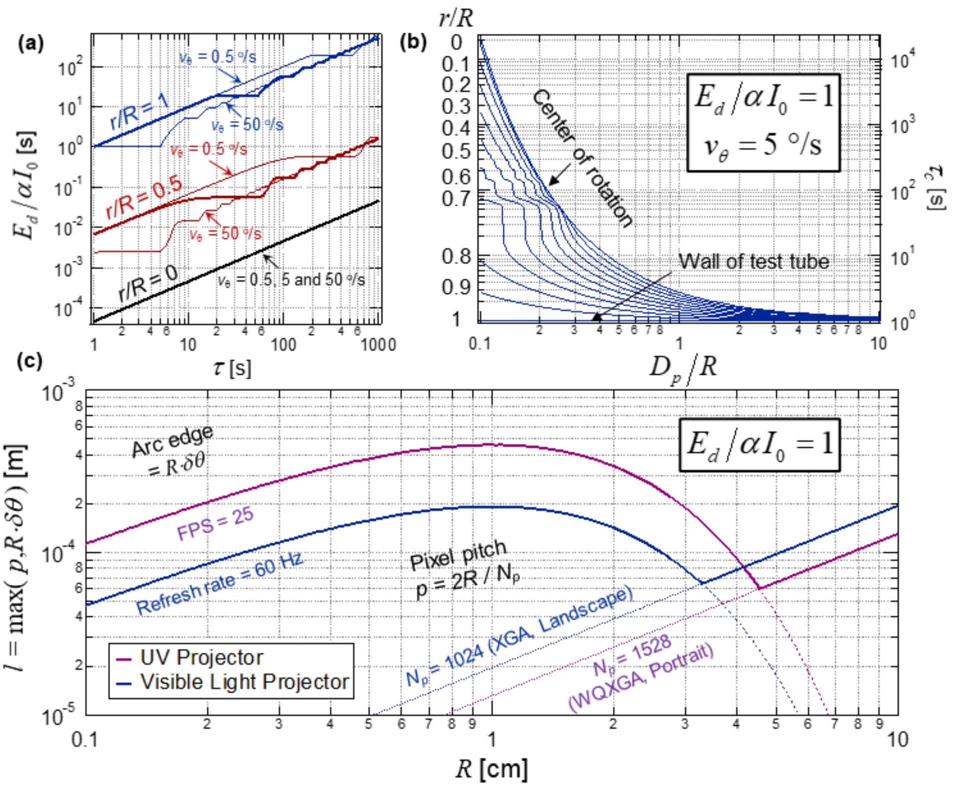

Supplementary Figure 5. Radial heterogeneity in dose build-up adversely impacts printing resolution and offers implications for precursor recipe and exposure time optimization. (a) The impact of rotation speed and radial position on dose build-up in a strongly absorbing medium (D_p/R = 0.1). (b) Effect of light attenuation (D_p/R) on curing time (τ_θ) given a target dose (E_d). A greater penetration depth favors the simultaneous consolidation of the entire workpiece. In contrast, decreasing medium transparency differentiates the curing time radially and results in inward-curing, i.e., the parts closer to the tube wall solidifies first, which benefits the printing of hollow structures. (c) The effective voxel size for a given photon supply-demand balance ($E_d/\alpha I_0$) is determined by the greater of two curves. For small workpieces, the voxel size is bottlenecked by the convex curve representing the limit set by curing time and refresh rate together. For larger workpiece, the projecting resolution and beam collimation becomes the limiting factor.

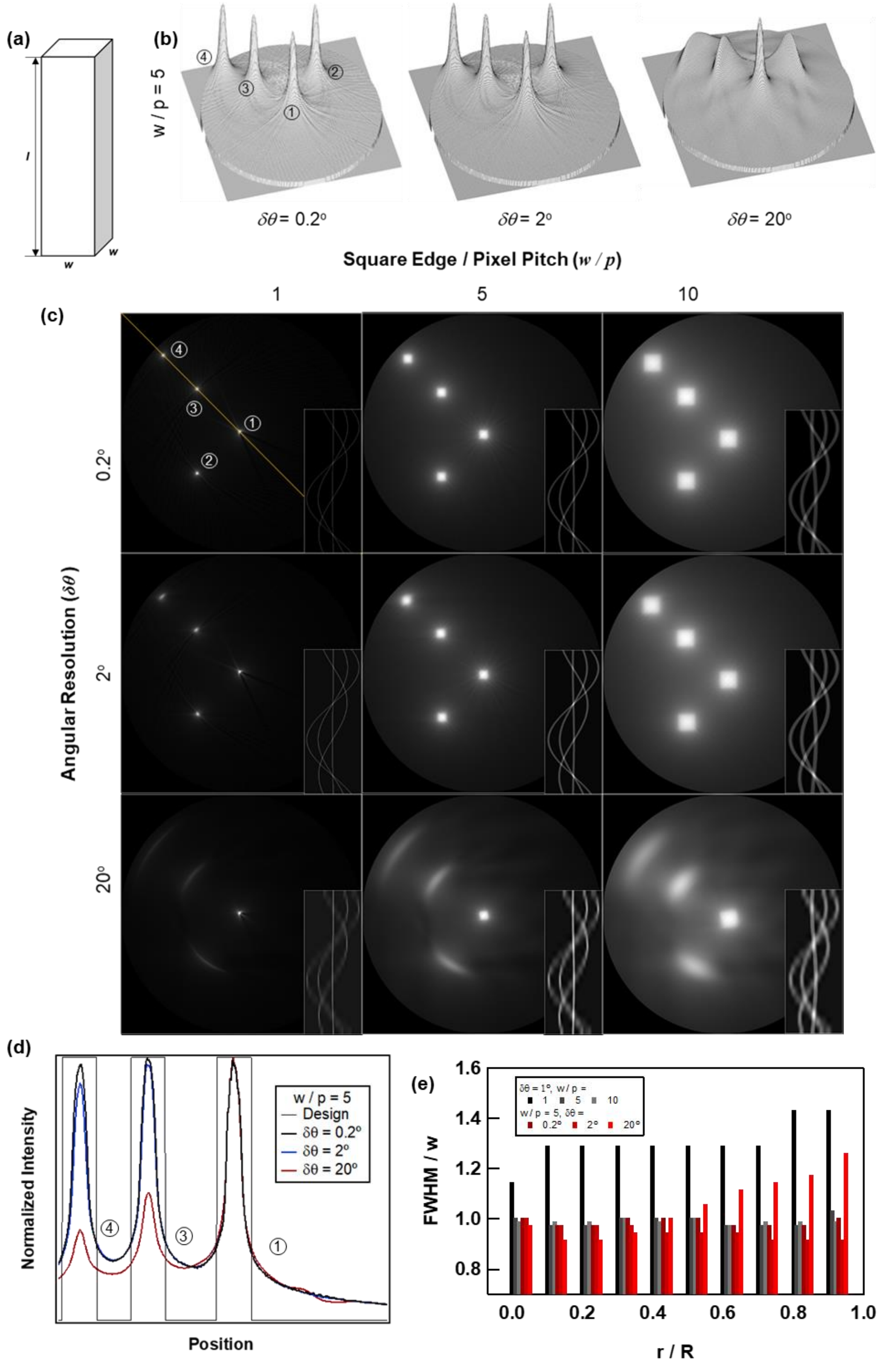

Supplementary Figure 6. Impulse response depicts the accuracy of dose delivery. (a) We mimicked the impulse response of the projector assembly by simulating the dose build-up

when a square post with edge *w* and intensity 1 was printed. (b) Perspective view of energy distribution in the printing plane indicates that an angular resolution of 2° reasonably reproduced features constituting 25 voxels. (c) The impact of angular and spatial resolution on feature spreading. A clear indication of insufficient angular refinement is that identical features are reproduced with a greater fidelity when closer to the rotation center. (d) At a large radial distance, a greater rotation speed smears the delivered dose over a longer arc, significantly lowering the average dose build-up in each voxel and thus deteriorating printing quality. (e) Effect of spatial and angular resolution on full width half maximum (FWHM) when a single square post was printed at different radial distance.

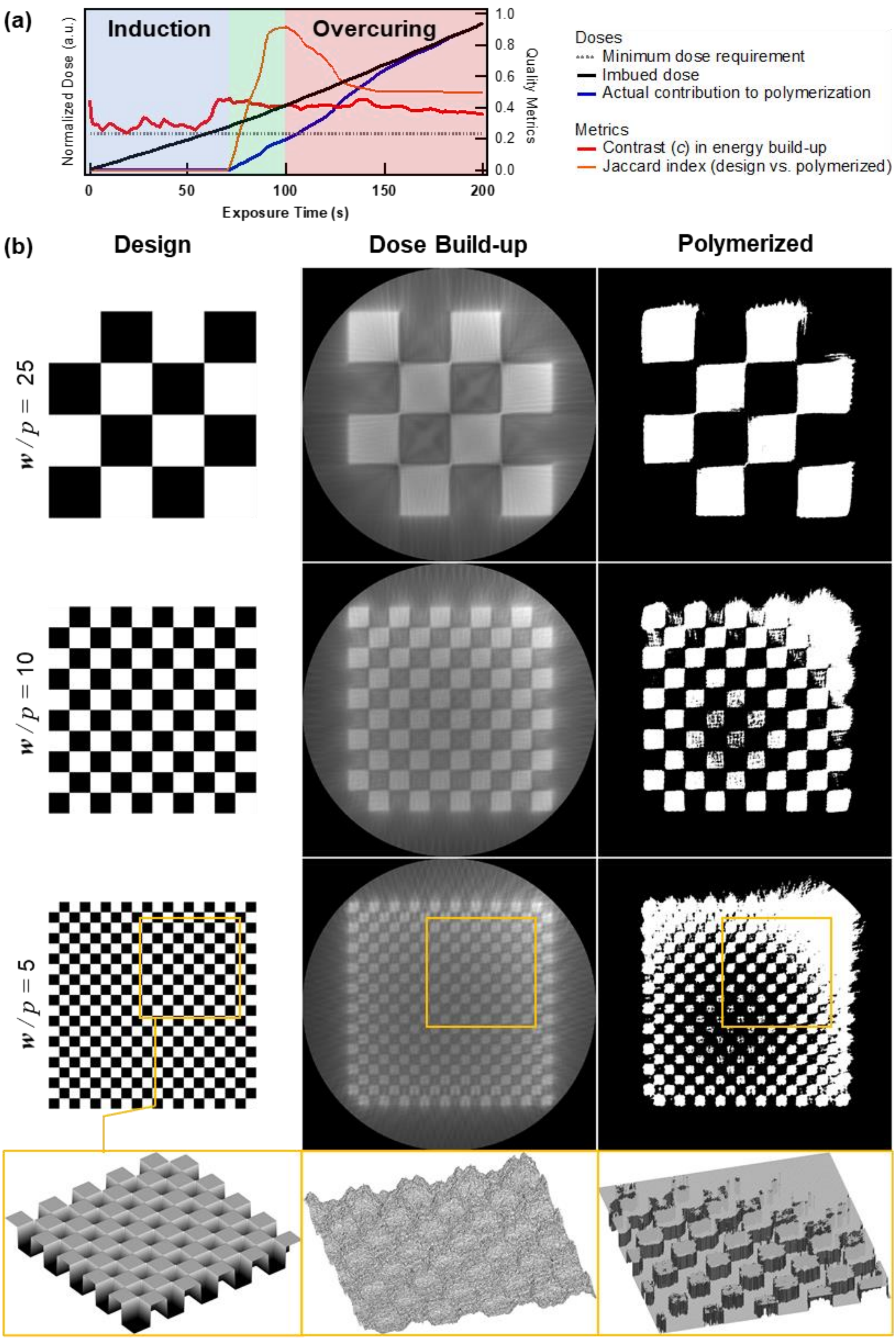

Supplementary Figure 7. Achieving sufficient contrast in dose build-up alone does not guarantee the printing quality when the nonlinear response of polymer precursor to incident light mismatches the rotation periodicity. (a) The three phases of TVP-based 3D printing. (b) The reproduction of design features of various sizes after 100 s exposure. Although the dose build-up resembles the intended feature, the polymer response introduces time-dependent artefacts. In particular, the shading effect stemming from local over-curing

(upper right corner) also causes local under-curing (lower left to the center) even if the overall delivered dose matches the minimum dose requirement.

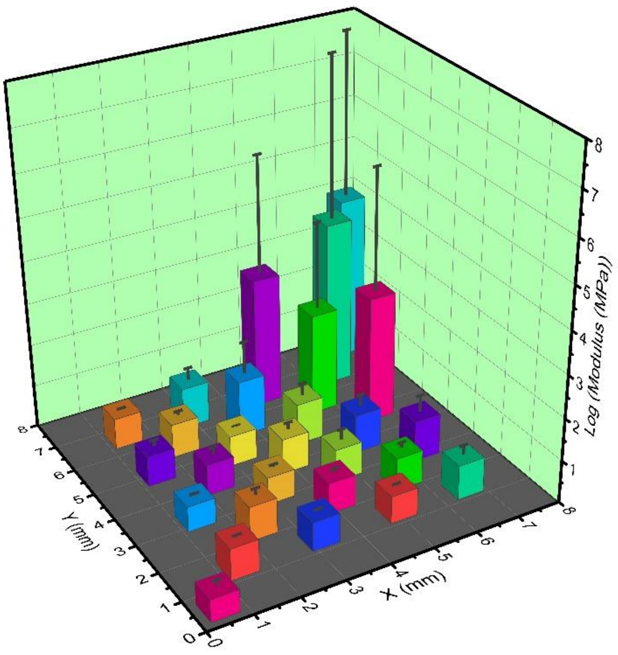

Supplementary Figure 8. 3D histogram of AFM-measured surface modulus distribution on a sample replicate of the one presented in Fig 1d. Force measurements were conducted on a 5 µm × 5 µm area (7 × 7 lattice) for each of the selected points (with the aid of camera that embed inside of the AFM to find the position). The numerical values are tabulated in Supplement Table 1.

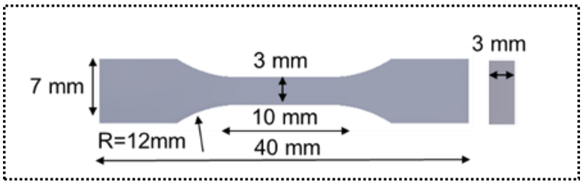

Supplementary Figure 9. Dimensions of the tensile test specimen (dogbone). The specimen was designed according to the standard of ASTM D-638 type IV. The specimen's overall length was down-scaled to 70% of the original design to fit into TVP's curing volume, while the gauge region was unchanged (10 mm).

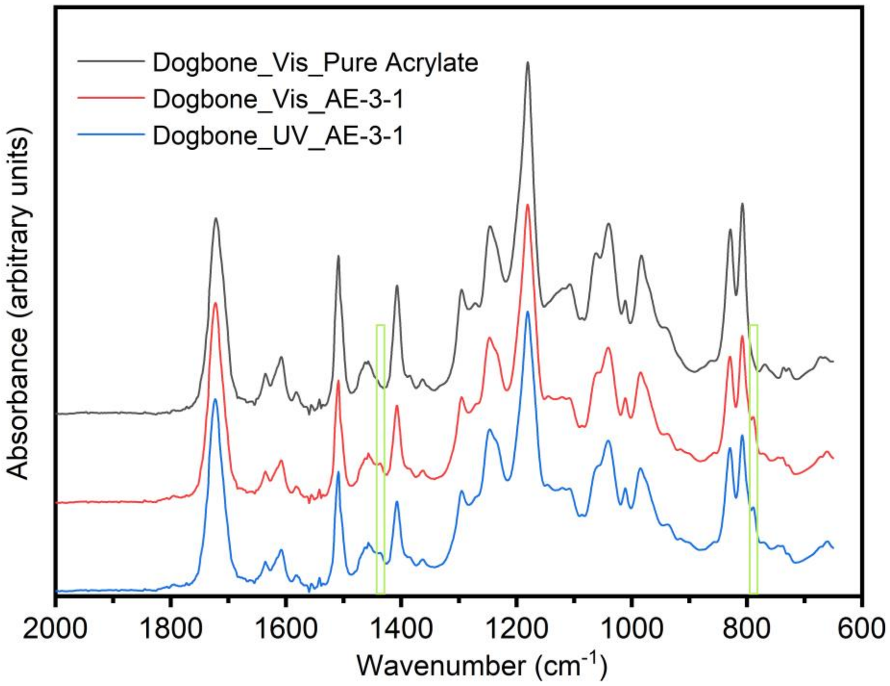

Supplementary Figure 10. Infrared spectrometry of the dogbone samples, printed using pure acrylate-based resin and resin AE-3-1 (without postprocessing). Green boxes mark the signature peaks of epoxy near 790 cm^{-1} and 1430 cm^{-1}.

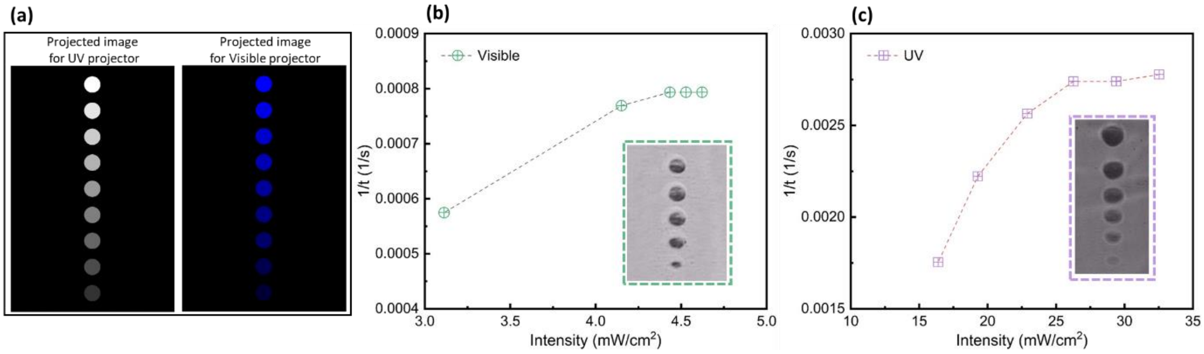

Supplementary Figure 11. Resin response characterization (AE-3-7). (a) The designed ball series projections for the UV projector and the Visible projector. (b – c) The relations between the irradiation intensity (varied with each ball) and the reciprocal of the curing time 1/t. During the printing process, within 30 minutes, 6 balls appeared sequentially with the UV exposure, while 5 balls appeared with the visible light. The curing time was not significantly affected by the intensity when the normalized gray value was greater than 200/255. The irradiation intensity was measured using an optical power meter (PM400+S120VC sensor, Thorlabs).

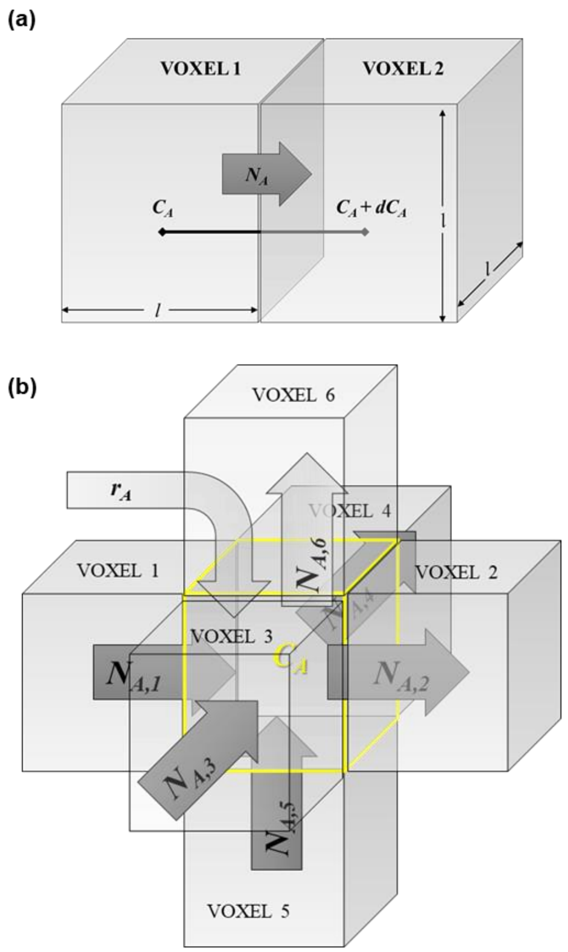

Supplementary Figure 12. Voxel-based discretization of the diffusion equation. (a) The Fickian diffusion was assumed to occur only at the interface between voxels and the mass flux was proportional to oxygen gradient. (b) The oxygen concentration was assumed uniform in each voxel. The mass balancing accounted for the diffusive fluxes across the six interfaces as well as the consumption of oxygen by irradiation as a pseudo zeroth-order photoreaction.

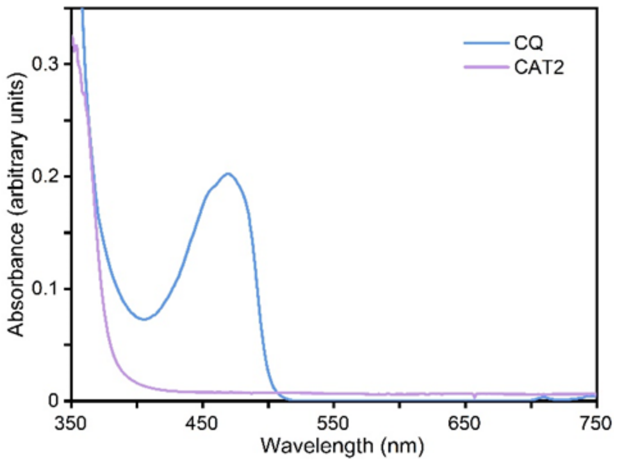

Supplementary Figure 13. Absorbance spectrum of CQ (blue line) and CAT2 (purple line)

Supplementary Tables 1-2

Supplementary Table 1. Exposure times and the delivered doses for the workpieces.

Workpiece	Confer	Material	Exposure time (s) /light dose (J)_Vis	Exposure time(s) /light dose (J)_UV
Binary grid composite workpiece	Fig.1a	AE-3-7	900/10	1224/52
Gray sheet composite structure	Fig.1c	AE-3-7	900/10	1224/39
Radially graded composite sample	Fig.1e	AE-3-7	900/10	1224/39
DTU logo	Fig.2a	AE-3-1	708/0.63	510/3.8
Nested structure	Fig.2b	AE-3-1	360/0.77	540/4.7
3D brain	Fig.2c	AE-3-1	406/0.52	300/3.2
Dogbone specimen	Fig.2e	AE-3-1	468/1.6	360/10
Ball (grey value 255)	Supplementary Figure 11	AE-3-7	1260/0.24	360/0.48
Ball (grey value 230)	Supplementary Figure 11	AE-3-7	1260/0.24	365/0.45
Ball (grey value 204)	Supplementary Figure 11	AE-3-7	1260/0.23	365/0.40
Ball (grey value 179)	Supplementary Figure 11	AE-3-7	1300/0.22	390/0.37
Ball (grey value 153)	Supplementary Figure 11	AE-3-7	1740/0.22	450/0.36
Ball (grey value 128)	Supplementary Figure 11	AE-3-7	-	570/0.39

Supplementary Table 2. Compressive modulus obtained using AFM-based nanoindentation (cf. Supplementary Figure 8)

Tested Points	XY position (approximate) corresponding to Supplementary Figure 8 (mm, mm)	Modulus (MPa)	Standard deviation (MPa)
1	(0.5, 0.5)	3.4	0.7
2	(1.4, 1.4)	5.5	1.1
3	(2.3, 2.3)	6.5	1.4
4	(3.2, 3.2)	4.4	1.1
5	(4, 4)	6.8	1.4
6	(4.8, 4.8)	9.4	1.9
7	(5.7, 5.7)	252	110
8	(6.6, 6.6)	8522	6332
9	(7.5, 7.5)	7728	7506
10	(1.4, 3.2)	3.9	0.9
11	(3.2, 1.4)	4.8	0.9
12	(1.4, 5)	5.1	1.4
13	(2.3, 4.1)	5.4	1.5
14	(4.1, 2.3)	5.4	1.2
15	(5, 1.4)	4.4	0.9
16	(1.4, 6.6)	5.4	1.1
17	(2.3, 5.7)	6.2	1.2
18	(3.2, 4.8)	5.2	1.0
19	(4.8, 3.2)	5.8	1.8
20	(5.7, 2.3)	5.8	1.4
21	(6.6, 1.4)	6.5	1.7
22	(3, 6.6)	9.1	2.0
23	(3.9, 5.7)	21.4	5.7
24	(5.7, 3.9)	8.0	1.9
25	(6.6, 3)	9.2	2.5
26	(4.8, 6.6)	1190	607
27	(6.6, 4.8)	980	775

Supplementary Notes 1-4

Supplementary Notes 1. Light Dose Estimation

1) Measuring the projector irradiation power related to different grey value image output (tabulated below), fit grey value x vs output light intensity I_x for interpolation, with non-negativity constraint.

Grey value (x)	Intensity_Vis_~455nm (mW/cm^2)	Intensity_UV_~365nm (mW/cm^2)
255	4.62	32.53
230	4.53	29.40
204	4.43	26.27
179	4.15	22.89
153	3.11	19.28
128	1.98	16.39
102	1.04	12.77
77		9.64
51		6.51
Estimated fit	$I_x = -0.0002x^2 + 0.0951x - 6.7158$	$I_x = 0.1287x - 0.1948$

2) Turning the projections into grayscale images and calculate the histogram of grey values for each frame in a projection sequence. According to the grey value distribution, it is doable to calculate the projection area A_x corresponding to the grey value x.

Define $A_x = \frac{C_x}{C_a} A$, In which

C_x ------ count of the pixel with the grey value x of selected projection

C_a ------ count of all the pixels of selected projection

A ------ projection area

3) The delivered light dose S is calculated as

$$S = \sum_{N} \sum_{0}^{255} I_x A_x \Delta t$$

In which, Δt ------ the residence time at each angle step

N ------ total number of frames

Taking the binary grid composite workpiece light dose calculation as an example.

- Vis projections: uniform image with gray value: 255, each projection dimension: 12*20 mm, total exposure time: 900s.
  Total light dose: $S_{vis} = 4.62 * 1.2 * 2 * 900 =$ 10 J

- UV projections: non-uniform image with graded gray value, each projection dimension: 11.3*20 mm (then area *A:* 2.3 cm^2), total exposure time: 1224s, number of projected frames *N*: 30600, each frame exposure time Δt : 0.04s. C_x and C_a were calculated by using industrial software Image J.
  Bring the data into the formula, then get total light dose:

$$S_{UV} = \sum_{30600}\sum_{0}^{255} I_x * A_x * \Delta t = \sum_{30600}\sum_{0}^{255}(0.1287x - 0.1948) * \frac{C_x}{C_a} A * \Delta t = 52 \text{ J}$$

Supplementary Notes 2. Impact of Projector Specifications on Voxel Size

A voxel in TVP takes the form of a fan-shaped cube (Fig. S4a) and has two characteristic lengths. In the plane perpendicular to the incident beam, the edge lengths (*p*) are determined by the pixel size of the projector:

$$p = \frac{2R}{N_p}, \qquad \text{(S1)}$$

in which *R* is the radius of the cylindrical curing volume and N_p is the effective number of horizontal pixels in the incident beam. N_p is typically determined by beam collimation and the specifications of the projector. In the tangential direction, the edge size is defined by $r \cdot \delta\theta$, in which *r* is the distance to rotation center and $\delta\theta$, the angular difference between two distinct projections. Here we consider the longest edge that limits the printing resolution, i.e. *r* = *R*. The voxel size, *l*, as a measure of printing quality, takes the value *l* = max (*p*, $R \cdot \delta\theta$). In forward projection, all unique projections can be found in [0,π), $\delta\theta$ is thus determined by

$$\delta\theta = \frac{\pi}{N_\theta}, \qquad \text{(S2)}$$

in which N_θ is the number of unique projections:

$$N_\theta = FPS \cdot \tau_c \qquad \text{(S3)}$$

FPS (frames per second) is capped by the refresh rate of the projector and τ_c, the induction period of a given point of interest *P*(x,y), is a function of (1) the intensity of incident beam (I_0, watt), (2) the curing dose of the polymer precursor (E_d, joule), (3) the absorptivity and attenuation coefficient of the precursor (α, dimensionless) and (4) the rotatory speed of the motor (v_θ, ^{o} s^{-1}). Assuming constant I_0 and α, the induction period is given by energy balancing:

$$E_d = \int_0^{\tau_c} \alpha I \cdot dt. \qquad \text{(S4)}$$

I is the light intensity at point *P* (Fig. S4b) and follows Beer-Lambert law:

$$\frac{dI}{dL} = -\alpha I. \qquad \text{(S5)}$$

Substituting the integral from into Eq. S4 and re-arrange gives

$$\frac{E_d}{\alpha I_0} = \int_0^{\tau_c} e^{-\alpha L} \cdot dt \,, \qquad \text{(S6)}$$

in which L is the length of attenuating light path $L = l_1 + l_2$

$$l_1 = -x\cos\theta - y\sin\theta \,, \qquad \text{(S7)}$$

$$l_2 = \sqrt{R^2 - \left(y\cos\theta - x\sin\theta\right)^2} \,, \qquad \text{(S8)}$$

$$\theta = v_\theta t \text{ , and} \qquad \text{(S9)}$$

$$\alpha \cdot D_p = 1 . \qquad \text{(S10)}$$

D_p is the penetration depth of light in the curing volume. Substitution gives:

$$\frac{E_d}{\alpha I_0} = \int_0^{\tau_c} e^{-\left(\frac{R}{D_p}\right)\cdot\left[\sqrt{1-\left(\frac{y\cos v_\theta t - x\sin v_\theta t}{R}\right)^2} - \left(\frac{x\cos v_\theta t + y\sin v_\theta t}{R}\right)\right]} \cdot dt \qquad \text{(S11)}$$

Eq. S11 gives the energy build-up profiles for points located at different distances to the rotation center (Figure S5a). These profiles were used as a qualitative guideline in optimizing exposure time. When light attenuation is not negligible, energy build-up ($E_d / \alpha I_0$) is sensitive to the periodicity of L. At the center (r/R = 0), L does not vary during rotation and the build-up is linear. Points located closer to the wall of the test tube (e.g., r/R = 0.5 and 1) cure faster as they receive stronger irradiance but are more prone to overexposure. It is thus desirable to adjust the rotation speed V_θ so that the plateau period during the building-up (e.g. τ = 20 – 50 s in the shown case) can be utilized to alleviate over-exposure. Combined with a lowered I_0, which shifts the blue and red profiles in Fig. S5a downwards, points at various r/R can be cured with approximately the same exposure time.

The induction period τ_c, which determines the tangential length of a voxel, can be computed from Eq. S11 once a desired dose E_d is defined. Fig. S5b shows the impact of the penetration depth (D_p, a measure of light attenuation in the curing volume) on the curing time. In practice, D_p is controlled by adjusting the concentration of photo-initiator ([PI]). Lowering [PI] (i.e., increasing D_p/R) can effectively ensure that the entire workpiece is polymerized at once. However, when printing hollow structures, a high [PI] can be employed to further differentiate the induction time dependence on r/R and to make sure that the shell (the parts closer to the wall of test tube) are cured while the inner portion of the rotating volume remains uncured. Fig. S5b also implies that the features closer to the rotation center tends to be reproduced with a greater fidelity in TVP because they receive more projections than features closer to the wall before the precursors are crosslinked.

The effective voxel size l reflects a concerted interplay among monomer properties (E_d, α), projector specifications (resolution, refresh rate, output power) and workpiece dimension (R). Fig. S5c shows an example of how l can be determined from the aforementioned factors. For small workpieces, l is determined by the tangential edge ($R\cdot\delta\theta$) and is limited by FPS. The convex shape stems from the fact that a larger workpiece requires a longer exposure time and

thus allows more projections to be delivered before a curing threshold is reached. At large *R*, *l* is determined by the length of the radial edge and thus the pixel pitch. An optimal *l* – R pair can be found where the two limiting curves intersect (e.g., *R* = 3.2 cm for blue light and 4.7 cm for UV in Fig. S5c). The demand and supply of photons is reflected in $E_d/\alpha I_0$, which shifts the FPS-dependent curves vertically. In Fig. S5c and on the right-hand side (RHS) of Eq. S11, the absorptivity was not accounted for. This choice was based on the consideration that, if the nonlinear response of polymer precursor is ignored, the energy build-up scales linearly with α everywhere in the distribution and does not change the shape of a curve. If not ignored, the point-based analysis presented here does not reflect the time-dependent shading effect of cured voxels (typically more absorptive) on the other voxels behind them, which is a predominant factor affecting printing quality. The shading effect is discussed in the *Contrast* section below.

Supplementary Notes 3. Impulse Response

The projector specifications set a hard limit on the smallest printable object. However, voxel size itself does not guarantee the fidelity of printouts. Voxels that are not intended to cure are irradiated inevitably as the incident light traverse the curing volume, leading to the blurring of features in naïve back-projection. This blurring is reflected directly in the shape of energy build-up. According to the central slice theorem, geometry reconstruction through back-projection is equivalent to drawing lines – one dimensional Fourier transform of the sinogram – across the origin of the spatial frequency domain, which results in a lower linear sampling rate away from the domain center. Sharp (high frequency) features are thus oppressed if no correcting filter is implemented before back projection. In this study, we do not use filters that produce negativity. It is thus of interest to investigate the effective printable features by simulating the impact of pixel pitch and angular resolution on impulse response.

We simulated the energy build-up of square post printing (Fig. S6a). The design contained four 2D square post functions (edge length *w*), one at the center of rotation (○,1, *r* (of the upper left corner of the square) = 0), one in the 3rd quadrant (○,2, *r/R* = 0.5) and two in the 2rd quadrant (○,3, *r/R* = 0.5 & ○,4, *r/R* = 0.9). The light attenuation and the nonlinear response of the polymer precursor were ignored. Both the shape and the altitude of dose distributions improved with increasing resolving capacity (Figs S6b,c). When multiple posts were printed simultaneously, a sufficiently small angular resolution was required to ensure the uniformity of build-up altitudes (Fig. S6d), otherwise features closer to the rim of the curing volume would show a strong "smearing" effect and be under-cured. An analysis of the full width half max (FWHM) for individually printed squares indicated that single voxel features were poorly reproduced when $\delta\theta = 1^o$ (Fig. S6e), with FWHM at least 10% greater than intended. In our simulation, five pixels were able to reasonably reproduce designed features, with FWHMs close to unity, measured at all *r/R* even at an angular resolution up to 2°. We conclude that, in a well collimated setup, the geometric fidelity of the energy build-up is mainly determined by projector specifications. The angular resolution, determined by the refresh rate of a projector, has not been a limiting factor in achieving small tangential voxel edge in our study. The printing quality of dual color TVP was thus ultimately controlled by the nonlinear response of the monomer solution to incident lights and hence, a careful

determination of exposure time.

Supplementary Notes 4. Contrast

Maintaining sufficient contrast during dose build-up is essential to achieving high geometric fidelity. A strategy we employed during trial-and-error in this study was to match the periodicity of rotation with an optimal exposure time at which the actual contribution of the incident light to polymerization equals the minimum dose requirement. A good matching was not always achievable, especially when the desired dose distribution was not binary. We explain this challenge in Fig. S7, in which the results were obtained using the *Simulator* module from the iterative sinogram computation routine (see *Methods*). We considered the nonlinear response of polymer to incident light and its effects on attenuation, absorption and shading. Further, we assumed that the diffusivity of oxygen was low in the viscous resin so that diffusion during the first 200 seconds of irradiation was negligible.

Fig. S7a shows the three phases of printing a checkerboard (*w / p* = 25). In the induction period (the blue regime), the curing volume remains homogeneous. The imbued dose increases linearly with exposure time while no polymerization was triggered. The slope reflects the output power of the projector. The contrast in dose build-up was calculated as

$$c = \frac{\bar{E}\left[E > 0.5\left(E_{\max} - E_{\min}\right)\right] - \bar{E}\left[E < 0.5\left(E_{\max} - E_{\min}\right)\right]}{\bar{E}\left[E > 0.5\left(E_{\max} - E_{\min}\right)\right] + \bar{E}\left[E < 0.5\left(E_{\max} - E_{\min}\right)\right]}, \qquad \text{(S12)}$$

in which E is the dose build-up in each voxel, $E_{\max}$ and $E_{\min}$ are the instantaneous maximum and minimum doses built in a single voxel, the bar notation indicates the averaging over all voxels that meet the criterion in the brackets. The contrast evolution reflects the periodicity of rotation, i.e., if the induction lasts longer than two or more rotation periods, one will observe a repetition of the evolution pattern of c with the same periodicity. In this example, the induction period was similar to the period of rotation (V_θ = 5 °/s) and thus no repetition was observed. During the curing phase (green), the critical dose was reached locally, and the workpiece started partial polymerization. The Jaccard index (orange curve) measures the similarity between the polymerized geometry and the original design and increases monotonically during this phase. The contrast evolution no longer repeats the previous pattern because of the heterogeneity caused by partial curing. The local polymerization changed the attenuation coefficient and the absorptivity of the workpiece in parts that were closer to the light source, which further exerted a shading effect on downstream voxels.

The shading effect is demonstrated in Fig. S7b, which shows the snapshots of *Dose Build-up* and *Polymerized* after 100 s, along with the original designs. The curing volume rotates clockwise with a period of 72 s. By 100 s, the upper right corner of the checkerboard received more doses than the rest of the curing volume and became more absorptive and attenuating. As a result, this corner became locally over-cured, while the region to the lower left of the rotation center remained under-cured in its shadow. This time-dependent deteriorative effect introduced by the nonlinearity of polymer response can be alleviated by matching the ideal exposure time with the rotation periodicity. The ideal exposure time is marked by the intersection between the minimum dose requirement and the actual contribution to polymerization (Fig. S7a, blue solid and black dash). In the shown case, the curing phase

would have concluded with a greater Jaccard index if the rotation period were set to 50 s. This imbalance between local dose delivery is more apparent for smaller features (Fig. S7b) and is nonetheless difficult to eliminate because partial curing is inevitable in the last rotation period before exiting the curing phase. This partial curing compromises the contrast in energy build-up (e.g., in Fig. S7a, *c* decreases in the green regime) and prevents a full reproduction of the original design.

After entering the overexposure phase (red), the Jaccard index drops quickly (for binary checkerboards, *J* stabilizes at 0.5). If a single polymer was formed, the curing volume will return gradually to a homogeneous state. The actual contribution to polymerization eventually merges with the imbued dose, at which point the entire curing volume solidifies.

Delineating the three phases of printing has important implications for the selection of printing parameters. For example, the difference between the imbued dose and the actual contribution as a function of time is attainable (e.g., using the *Simulator* module). The minimum dose requirement stems from energy balancing and is proportional to the volume of a workpiece as well as the curing threshold of the chosen precursor. The optimal exposure time and therefore the rotation speed can thus be identified. Also, because the maximum output power of a projector is a constant (the slope of imbued dose vs. time) while the minimum dose varies for each slice in the vertical direction, an optimal projecting sequence would require the gray values of each projection be rescaled vertically slice by slice.
