# Supplementary material for: Stiffness control in dual color tomographic volumetric 3D printing
Source: Nat Commun. 2022 Jan 18;13:367. doi: 10.1038/s41467-022-28013-4 (PMC8766567; doi:10.1038/s41467-022-28013-4)
Supplement: Supplementary file 2 — Description of Additional Supplementary Files [file 41467_2022_28013_MOESM2_ESM.pdf]

Description of Additional Supplementary Files

File Name: Supplementary Movie 1.mp4
Description: Printing of the DTU Logo

File Name: Supplementary Movie 2.mp4
Description: Printing of a Voronoi Tower

File Name: Supplementary Movie 3.mp4
Description: Printing of an Einstein Bust Figurine

File Name: Supplementary Software.zip
Description:
SC1. Iterative Sinogram Computation
SC2. Print simulation with inhibitor diffusion

File Name: Supplementary Information.pdf
Description: Supplementary Information, including
Supplementary Figures 1-13
Supplementary Tables 1-2
Supplementary Notes 1-4

File Name: Source Data.xlsx
Description: Compilation of all data generated or analysed in this study
